# Australian shellfish ecosystems: Past distribution, current status and future direction

**DOI:** 10.1371/journal.pone.0190914

**Published:** 2018-02-14

**Authors:** Chris L. Gillies, Ian M. McLeod, Heidi K. Alleway, Peter Cook, Christine Crawford, Colin Creighton, Ben Diggles, John Ford, Paul Hamer, Gideon Heller-Wagner, Emma Lebrault, Agnès Le Port, Kylie Russell, Marcus Sheaves, Bryn Warnock

**Affiliations:** 1 The Nature Conservancy Australia, Carlton, Victoria, Australia; 2 TropWATER (Centre for Tropical Water and Aquatic Ecosystem Research), James Cook University, Townsville, Queensland, Australia; 3 Primary Industries and Regions South Australia, Adelaide, South Australia, Australia; 4 Centre of Excellence in Natural Resource Management, University of Western Australia, Albany, Western Australia, Australia; 5 Institute of Marine and Antarctic Studies, University of Tasmania, Hobart, Tasmania, Australia; 6 DigsFish Services, Banksia Beach, Queensland, Australia; 7 School of Biosciences, The University of Melbourne, Parkville, Victoria, Australia; 8 Victorian Fisheries Authority, Queenscliff, Victoria, Australia; 9 Fisheries NSW, NSW Department of Primary Industries, Port Stephens, New South Wales, Australia; 10 College of Science and Engineering, James Cook University, Townsville, Queensland, Australia; Florida Atlantic University, UNITED STATES

## Abstract

We review the status of marine shellfish ecosystems formed primarily by bivalves in Australia, including: identifying ecosystem-forming species, assessing their historical and current extent, causes for decline and past and present management. Fourteen species of bivalves were identified as developing complex, three-dimensional reef or bed ecosystems in intertidal and subtidal areas across tropical, subtropical and temperate Australia. A dramatic decline in the extent and condition of Australia’s two most common shellfish ecosystems, developed by *Saccostrea glomerata and Ostrea angasi* oysters, occurred during the mid-1800s to early 1900s in concurrence with extensive harvesting for food and lime production, ecosystem modification, disease outbreaks and a decline in water quality. Out of 118 historical locations containing *O*. *angasi*-developed ecosystems, only one location still contains the ecosystem whilst only six locations are known to still contain *S*. *glomerata*-developed ecosystems out of 60 historical locations. Ecosystems developed by the introduced oyster *Crasostrea gigas* are likely to be increasing in extent, whilst data on the remaining 11 ecosystem-forming species are limited, preventing a detailed assessment of their current ecosystem-forming status. Our analysis identifies that current knowledge on extent, physical characteristics, biodiversity and ecosystem services of Australian shellfish ecosystems is extremely limited. Despite the limited information on shellfish ecosystems, a number of restoration projects have recently been initiated across Australia and we propose a number of existing government policies and conservation mechanisms, if enacted, would readily serve to support the future conservation and recovery of Australia’s shellfish ecosystems.

## Introduction

Oysters and mussels are ecosystem engineers [1] that create, modify and maintain habitat for a range of other species at a system-wide scale [2, 3]. When occurring in dense aggregations they form reef and bed structures comprised of both living assemblages and/or dead shell accumulations. These bivalve created ecosystems contain unique biological communities and vary in physical nature from consolidated structures with a high vertical profile (often termed reefs) to low profile structures with little differentiation in relief from their surrounds (aggregations or beds) and also include shell-rich muddy bottoms (accumulations) [3–5]. For the purpose of this study, we define shellfish ecosystems as: intertidal or subtidal three-dimensional biogenic structures, formed primarily by high densities of oysters and/or mussels and their associated biological communities. We include shellfish ecosystems with high or low vertical profiles (i.e., reefs and beds) created on otherwise soft sediments or rocky areas. Shellfish ecosystems provide a range of ecosystem services such as food provision [6, 7], habitat for fish and invertebrates [2, 8–10] water filtration [11–13], fish production [14] and shoreline protection [15, 16]. Formally covering vast areas of coastal waters in both temperate and tropical regions, shellfish ecosystems have been decimated globally, with over 85% lost or severely degraded through a combination of actions including overfishing, destructive fishing practices (e.g., dredging), water pollution and disease [6, 13, 17].

The loss of shellfish ecosystems, in addition to the loss and degradation of other important marine ecosystems such as seagrass meadows [18], kelp forests [19, 20], saltmarshes [21] and mangrove forests [22], is a key driver in the long-term degradation of estuarine and coastal waterways, contributing to declines in water quality, fish production, blue carbon and coastal protection [23]. These losses may negatively affect the economic and social wellbeing of coastal human populations reducing the productivity of wild harvest fisheries, increasing pollution risks to coastal communities and industries (e.g. aquaculture) and by increasing the cost of protecting coastal assets with non-natural (‘built’) infrastructure [24–26]. The degradation of coastal ecosystems also contributes to the release of stored carbon, further exacerbating climate change and increasing coastal risks associated with more frequent and intense storms, sea level rise and ocean acidification [27].

Despite recent efforts to improve the protection of coastal ecosystems through international conventions and treaties (e.g. Convention on Biological Diversity and the Ramsar Convention), there is growing recognition that protection as a means to conserve coastal ecosystems and their ecosystem services is not enough and that active repair and restoration is required for many marine ecosystems [28, 29]. Efforts to restore shellfish ecosystems and their ecological function are most advanced in the United States, where hundreds of oyster reefs have been restored over the last 15–20 years [12, 30–32]. These efforts have led to the return of ecological functions and social and economic benefits such as job creation and fish production [33, 34]. Shellfish ecosystem restoration has been suggested as a mechanism for bringing back lost ecological functions in Australian estuaries and coastal waters [35, 36]. However, ecological knowledge and examples of shellfish ecosystem restoration and the underpinning science relating to the ecology, structure and distribution of shellfish ecosystems are rare outside of the United States and particularly so in the Southern Hemisphere, where only a handful of studies have documented their distribution and decline [7, 8, 37–42].

Here we analyse the available evidence on Australian shellfish ecosystems and synthesize current knowledge, with the objectives of: 1) identify common ecosystem-forming species 2) quantify their past and present distributions, 3) review causes for shellfish ecosystem decline and 4) identify the current state of scientific knowledge including Indigenous use for Australian shellfish ecosystem-forming species. From this body of knowledge, we also highlight knowledge gaps and propose several recommendations which, if enacted, could help improve the future management of shellfish ecosystems. Our study can also serve as an example of a potential starting point for other regions where information on shellfish ecosystems may currently be scarce or unknown.

## Methods

### Identifying ecosystem-forming bivalve species

A primary objective of this study was to identify common ecosystem-forming species in light of a general underappreciation of shellfish ecosystems as a unique marine ecosystem in Australia, highlighted previously by Alleway and Connell [37]. For instance, despite numerous historical references to shellfish ecosystems in state government reports pre-1950 (see below), modern state and federal environmental government agencies do not typically recognise shellfish ecosystems as a marine ecosystem type. Consequently, government-initiated marine ecosystem mapping programs rarely map shellfish ecosystem extent or provide definitions of ecosystem type. We therefore used a process of expert elicitation through members of the Australian Shellfish Reef Restoration Network (http://www.shellfishrestoration.org.au) to nominate and evaluate whether candidate bivalve species should be considered ecosystem-forming in an Australian context. We evaluated species against two predefined criteria: 1) confirm to the definition of shellfish ecosystems (as defined in the Introduction and [38]) and 2) sufficient information in the published and/or grey literature to infer classification as an ecosystem-forming species. We limited our assessment to those species that formed primary biogenic ecosystem on soft sediments and/or rocky areas rather than those which may form dense aggregations only on plants or animals. We also excluded species that predominantly reside in sediments (endobenthic), such as Mesodesmatidae (surf clams), Cardiidae (cockles), Donacidae (pipis) and some species of mussels (*Xenostrobus inconstans*).

### Knowledge review: Indigenous and colonial use, historical harvest and regulation, past extent and current distributions

In 2015, the Australian National Environmental Science Programme Marine Biodiversity Hub supported a series of expert workshops and regionally focused reports which aimed to summarise Indigenous and early colonial uses of shellfish ecosystems including historical harvest, food consumption and regulation. This body of work also aimed to provide a cursory review of ecosystem-forming species and their past and current extents [workshops and report findings summarised in 35]. We used this research as the basis of our analysis and identified further evidence through: 1) a review of published and grey data sources likely to have information related to the nominated ecosystem-forming species 2) an appraisal of available wild oyster and mussel harvest records and newspaper accounts, 3) identification of historical or current bivalve aquaculture sites and 4) identification of localities where names are related to oysters and mussels (e.g. Oyster Harbour, Limeburners Bay). Methods undertaken to conduct 1–4 above are described below.

To conduct the literature review, we identified published journal articles for all nominated ecosystem forming species using the database Scopus (https://www.scopus.com/) supplemented by Google Scholar (https://scholar.google.com/). The search terms: ‘Australia’, ‘oyster’, ‘mussel’ and each of the species names listed in Table 1 were used to build a bibliography of all papers published up until March 2016 (S1 Table). We reviewed the primary objectives and results of each paper and categorised them according to several, non-discrete groups related to shellfish ecosystem ecology, aquaculture or both.

**Table 1:** Description of Australian ecosystem-forming bivalve species.

Species	Distribution^a^	Habit	Comments
***Brachidontes erosus*** (Lamarck 1819) Eroded mussel	TAS, VIC, SA, WA	Intertidal to shallow subtidal on hard substrates	No recorded fishery or aquaculture within Australia. May only form ecosystems occasionally or in association with other ecosystem-forming shellfish species.
***Brachidontes rostratus*** (Dunker 1857) Beaked mussel	SA,VIC, TAS, southern NSW	Intertidal to shallow subtidal on hard substrates	No recorded fishery or aquaculture within Australia. May only form ecosystems occasionally or in association with other ecosystem-forming shellfish species.
***Crassostrea gigas*** (Thunberg 1793) Japanese oyster, Pacific oyster (introduced)	TAS, VIC, SA, NSW	Intertidal to shallow subtidal forming reefs on hard substrates	Introduced from Japan in 1947 for aquaculture. Aquaculture current in TAS, SA and NSW. Wild populations likely growing in extent in NSW, TAS and SA.
***Isognomon ephippium*** (Linnaeus 1758) leaf oyster, rounded toothed pearl shell	NSW, QLD, northern WA.	Intertidal forming beds on mudflats, sandy bottoms and hard substrates	No recorded fishery or aquaculture within Australia. Previously not recorded as ecosystem-forming.
***Limnoperna (Xenostrobus) pulex*** (Lamarack 1819) Flea mussel	WA, VIC, TAS, NSW	Intertidal forming beds on hard surfaces	No recorded fishery or aquaculture within Australia. Typically forms small beds on exposed intertidal platforms.
***Malleus meridianis*** (Cotton 1930) Southern hammer oyster	WA, SA	Subtidal on broken rubble and sheltered habitats	No recorded fishery or aquaculture within Australia. May only form ecosystems occasionally or in association with other ecosystem-forming shellfish species, particularly in SA.
***Mytilus* (*edulis*) galloprovincialis** (Lamarck 1819) blue mussel, bay mussel	southwest WA, SA, VIC, TAS, NSW	Intertidal and subtidal to 10 m forming beds on hard surfaces and sandy bottoms	Historic dredge fishery in VIC. Aquaculture current in TAS, NSW, VIC, SA and WA. Common ecosystem-forming species.
***Ostrea angasi*** (Sowerby 1871) Angasi oyster, flat oyster, mud oyster, Port Lincoln oyster	NSW, VIC, TAS, SA, WA	Subtidal to 30 m forming reefs on hard substrates and soft sediments	Substantial dredge fishery occurred from mid-1800s to mid-1900s in all states. Aquaculture current or previously attempted in TAS, NSW, VIC, SA and WA. Common ecosystem-forming species.
***Pinctada albina sugillata*** (Reeve 1857) pearl oyster	NT, QLD, and upper Spencer Gulf, SA	Low intertidal to at least 50 m forming beds on hard substrates	No recorded fishery or aquaculture within Australia. Forms ecosystems only occasionally in upper Spencer Gulf, SA, possibly near Groote Eylandt, NT and southern QLD.
***Pinna bicolour*** (Gmelin 1791) Razor fish, razor clam	WA, NT, QLD, NSW	Intertidal and subtidal to 10 m on sand and broken bottoms	No recorded fishery or aquaculture within Australia. May only form ecosystems occasionally (particularly in SA) or in association with other ecosystem-forming shellfish species.
***Saccostrea cucullata*** (Born 1778) Milky oyster, coral-rock oyster	QLD, NT, WA	Intertidal forming reefs on hard substrates including mangroves and dead coral	Previously formed an important local hand harvest fishery in central and southern QLD. Current small-scale harvest and aquaculture trials. Common ecosystem-forming species.
***Saccostrea echinata*** (Quoy & Gaimard 1835) Black lip oyster	NT, QLD	Intertidal and shallow subtidal hard surfaces	No recorded fishery or aquaculture within Australia. May only form ecosystems occasionally or in association with other ecosystem-forming shellfish species
***Saccostrea glomerata*** (Gould 1850) rock oyster	Southern QLD, NSW, far eastern VIC	Intertidal forming reefs on hard and soft substrates. Historical records describe subtidal reefs to 8 m	Dredge and hand fishery on the east coast of Australia from early 1800s, aquaculture in NSW, QLD and WA. Common ecosystem-forming species.
***Trichomya hirsuta*** (Lamarck 1819) hairy mussel	SA, VIC, northern TAS, NSW, QLD.	Low intertidal on hard substrates often in bands below oysters to at least 3.5 m	No recorded fishery or aquaculture within Australia. May only form ecosystems occasionally or in association with other forming shellfish species.

^a^ NT = Northern Territory, WA = Western Australia, SA = South Australia, VIC = Victoria, TAS = Tasmania, NSW = New South Wales, QLD = Queensland.

We considered early explorer accounts from 1770 and historical fisheries records maintained by state government fishery agencies from ~1850 to October 2015 for all coastal states (excluding the Northern Territory). We also searched historical newspaper articles for references to wild oyster or mussel fishing, harvesting, and/or dredging from 1800 to 1950 using the online database *Trove*- a national online catalogue of over 124 million newspaper articles maintained by the National Library of Australia (http://trove.nla.gov.au). We searched for locality names containing Oyster, Mussel or Limeburner using the online database Gazetteer of Australian Place Name Search (www.ga.gov.au) and obtained data on shellfish aquaculture locations from relevant state government agencies.

To determine the past and current extent of shellfish ecosystems we followed the methods of Kirby [7], Zu Ermgassen et al. [13] and Alleway and Connell [37], where in the absence of historical quantitative data on shellfish density or extent (e.g. commercial harvest records or ecological surveys), we applied a ‘threshold approach’ as a proxy for historical shellfish ecosystem extent. This method uses the presence of commercial shellfish harvesting which occurred prior to 1950 as a proxy for high oyster and mussel density, thus indicating a strong likelihood of a shellfish ecosystem occurring at the identified locality. We attributed historical commercial fishing (and thus the presence of a shellfish ecosystem) to a location if a primary or secondary source was able to provide a description of: (1) commercial shellfish harvesting occurring at a specified location (2) a method of extraction (e.g. hand harvest, oyster dredge) and, where possible, (3) the amount of extraction (e.g. bags, bushels, number of oysters or mussels, tonnes).

To enable a comparison of historical and current extents, we defined the minimum, comparable geographic unit across time as a single reef or bed covering > 1 ha in size, or, a system of reefs or beds covering > 1 ha in size which we collectively termed ‘reef system’. We selected 1 ha reef systems as a comparable unit of measurement based on: 1) the assumption that historical commercial fishing was unlikely to occur in areas where ecosystem extent was less than 1 ha in size (as described above, constituting a minimum harvestable threshold), 2) a review of the only commercially-harvested natural shellfish ecosystem remaining in Australia which includes the shellfish ecosystem covering a similar spatial scale [40] and 3) general consensus amongst workshop participants that 1 ha was a conservative yet comparable geographic unit of measurement to assess historical and current shellfish ecosystem extent. Due to the lack of available data, we were unable to use a smaller unit of measurements such as those normally used to define shellfish ecosystems (i.e. areas typically consisting multiple reef or bed patches, with at least some of the patches being larger than 5 m^2^ and dominated by at least 25% cover of live shell matter [38]).

We therefore consider our analysis of the decline in distribution for *O*. *angasi* and *S*. *glomerata* to be conservative, as we only measure decline in extent for localities where the shellfish ecosystem occurred in areas > 1 ha in size. It is highly plausible that shellfish ecosystems occurred at smaller scales (i.e. > 5m^2^ but smaller than < 1 ha in extent) in many other historical localities not identified in our study. Our analysis is therefore a comparison of the number of *localities* (i.e. bays, estuaries) across Australia that contained a minimum area of shellfish ecosystem > 1 ha in size, prior to 1950 compared to 2015. This constitutes an assessment of extent of occurrence at the locality-scale rather than total area of occupancy within and across localities.

We used the presence of modern shellfish aquaculture as a second line of evidence for identifying locations with historical shellfish ecosystems, since historical accounts often described the development of shellfish aquaculture in the same locations as historical harvesting [35 and refs there in, 43, 44]. We used locality names containing ‘Oyster*’, ‘Mussel*’ and ‘Limeburner*’ (with * denoting a wildcard for the associated geographic feature e.g., harbour, bay, estuary) as a third line of evidence to identify localities where shellfish abundance was clearly a historical distinguishing feature of the local geography or as further evidence that commercial fishing occurred in close proximity (oyster shells were burnt in kilns to make lime used in building materials, hence locality name of Limeburners). We attribute a locality name to a species only when a single, commercially harvested, ecosystem-forming species occurred at that location (i.e. *O*. *angasi* in southern Western Australia, South Australia, Victoria or Tasmania). Where more than one commercially harvested species occurred in a region (i.e. *S*. *glomerata* and *O*. *angasi* in New South Wales, we assumed the locality was named after the species primarily harvested and sold at market (*S*. *glomerata* for New South Wales).

We note not all states had comparable amounts of data, for instance, early government fisheries records and maps from New South Wales are largely absent prior to September 1882, due to a fire at the Garden Palace Library in Sydney destroying all historical records. The current locations of *O*. *angasi* and *S*. *glomerata* reefs were identified by expert elicitation [35] and from the scientific and grey literature according to the criteria described above (i.e. localities with ecosystem extent > 1 ha).

## Results and discussion

### Ecosystem-forming species

**Fig 1:**
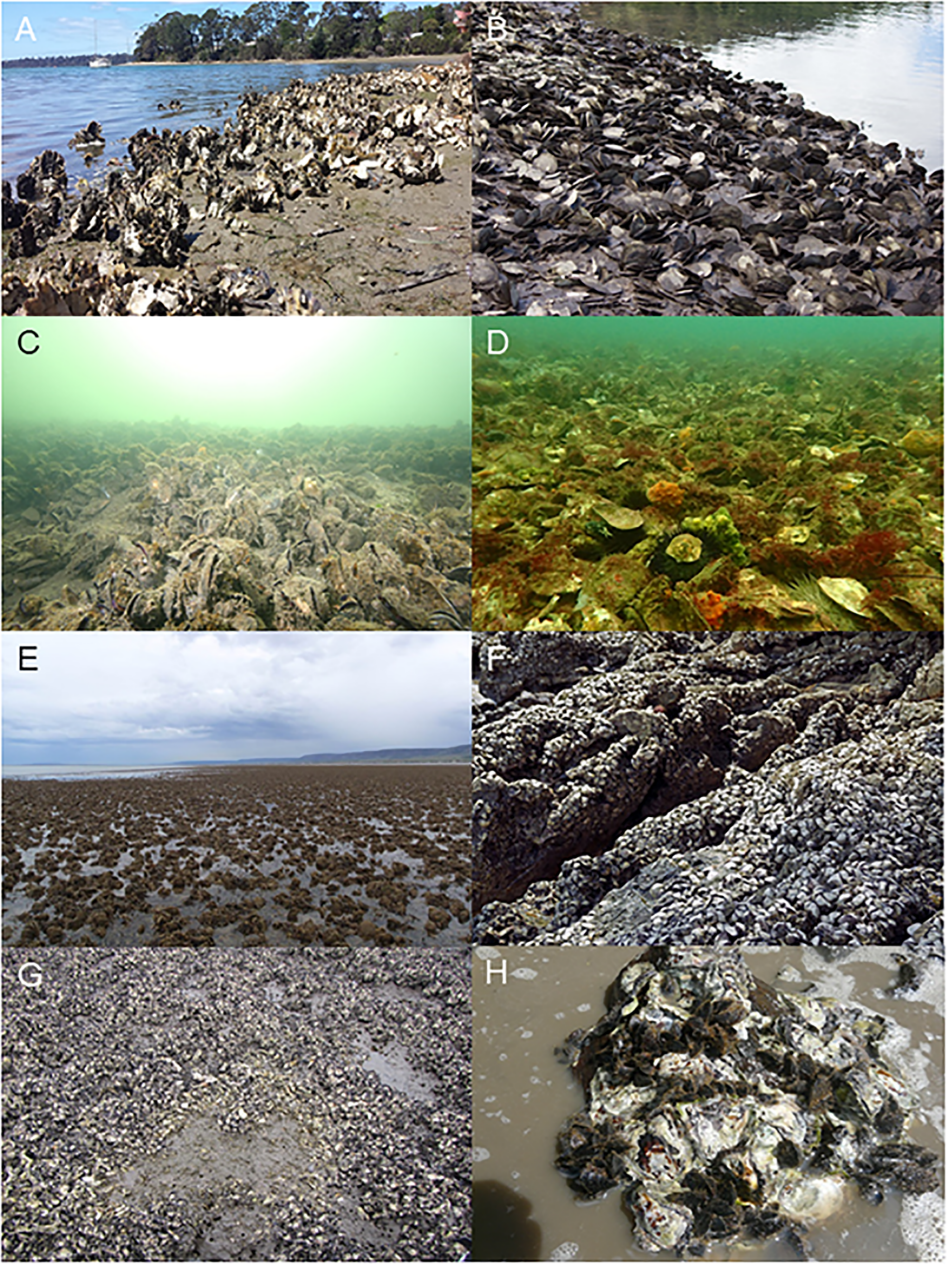
Examples of form and structure of Australian shellfish ecosystems. A = *Crassostrea gigas*, B = *Isognomon ephippium*, *C = Mytilus galloprovincialis D = Ostrea angasi*, *E = Pinctada albina sugillata*, F = *Saccostrea cucullata*, G = *Saccostrea glomerata*, *H = Trichomya hirsuta*. Reproduced from [35].

Nine oyster and five mussel species were identified as clearly forming or likely to form defined reef or bed ecosystems (Fig 1, Table 1) including *Crassostrea gigas* (Pacific oyster) an oyster introduced from Japan in 1947 for the purposes of aquaculture. Generally, shellfish ecosystems occur in bays, estuaries and nearshore coastal waters in both tropical and temperate regions across every state within Australia including the Northern Territory (Table 1). With the exception of *O*. *angasi* and *Pinna bicolor*, which can form reefs or beds down to a depth of tens of metres in oceanic waters, and *S*. *glomerata* and *Mytilus (edulis) galloprovincialis*, which historically formed subtidal reefs down to depths of eight meters or more [39, 45], all other species are likely to form reefs or beds in the intertidal or shallow subtidal regions. We identified several species *Pinctada albina sugillata*, *Saccostrea cucullata*, *Isognomon ephippium* and *Trichomya hirsuta* yet to be reported in the literature as ecosystem-forming with previous studies only considering the most common and widely distributed species (*O*. *angasi*, *S*. *glomerata*, *C*. *gigas and M*. *galloprovincialis*). The degree to which *Brachidontes erosus*, *Brachidontes rostratus*, *Limnoperna pulex* and *Saccostrea echinata* form discernible ecosystems which conform to standard shellfish ecosystem definitions (e.g. [3, 38]) is not yet clear, whilst *P*. *bicolour and Malleus meridianis* are likely to only form shellfish ecosystems in South Australia’s unique gulf systems [46, 47]. We suggest clarifying the degree to which the lesser known species form unique ecosystems should be the focus for future research.

### Literature review and locality information for ecosystem-forming species

We identified 223 scientific peer-reviewed publications on the 14 identified bivalve species with the majority of studies focused on those species with the highest commercial value, i.e. *S*. *glomerata* (113) and *C*. *gigas* (50) (Table 2, S1 Table). In contrast, very few research papers have been produced for the other species: *M*. *galloprovincialis* (27), *O*. *angasi* (16), *P*. *bicolor* (11) *P*. *albina sugillata* (11), *S*. *cucullata* (8), *I*. *ephippium* (6), *L*. *pulex* (6) and *T*. *hirsuta* (4), *B*. *erosus (3)*, *B*. *rostratus* (3), *M*. *meridianis* (2) and *S*. *echinata* (2). This body of research has largely focused on the ecology and distribution of natural oyster populations (47%) including 18 studies on the historical ecology and distribution of shellfish ecosystems. Subjects relating to shellfish aquaculture and animal husbandry were also common (42%). Comparatively less research has focused on the conservation and protection of natural reef ecosystems (5%) or their ecosystem services (5%), information which is critical for understanding their conservation status and in building the case for recovery.

**Table 2:** Summary of the literature review on ecosystem-forming bivalve species by subject area. Papers were assigned to more than one category if they covered multiple subjects.

Subject	Category	No. of Papers	% Total
*Ecosystem services of oysters and reefs*	Shoreline protection (SP)	0	5
	Water filtration (WF)	3	
	Habitat value (e.g. fish) (HV)	22	
*Conservation*	Policy and management (natural populations) (NPM)	7	6
	Restoration ecology & history (RE)	8	
	Protection (PT)	11	
*Ecology of (natural) oysters and reefs*	Biological description (BD)	19	47
	Climate change (CC)	7	
	Natural reproduction (NR)	22	
	Biodiversity (BI)	22	
	Invasive species (IS)	16	
	Historical ecology (HI)	18	
	Natural distribution (ND)	53	
	Health (EH)	71	
*Aquaculture and animal husbandry*	Depth effect (DE)	7	42
	Policy & management (aquaculture populations) (APM)	7	
	Feeding (FE)	9	
	Marketing (MK)	18	
	Cage optimization (CO)	22	
	Aquaculture reviews (AR)	27	
	Health (AH)	28	
	Culture methodology (CM)	33	
	Trait selection (TS)	60	

**Fig 2:**
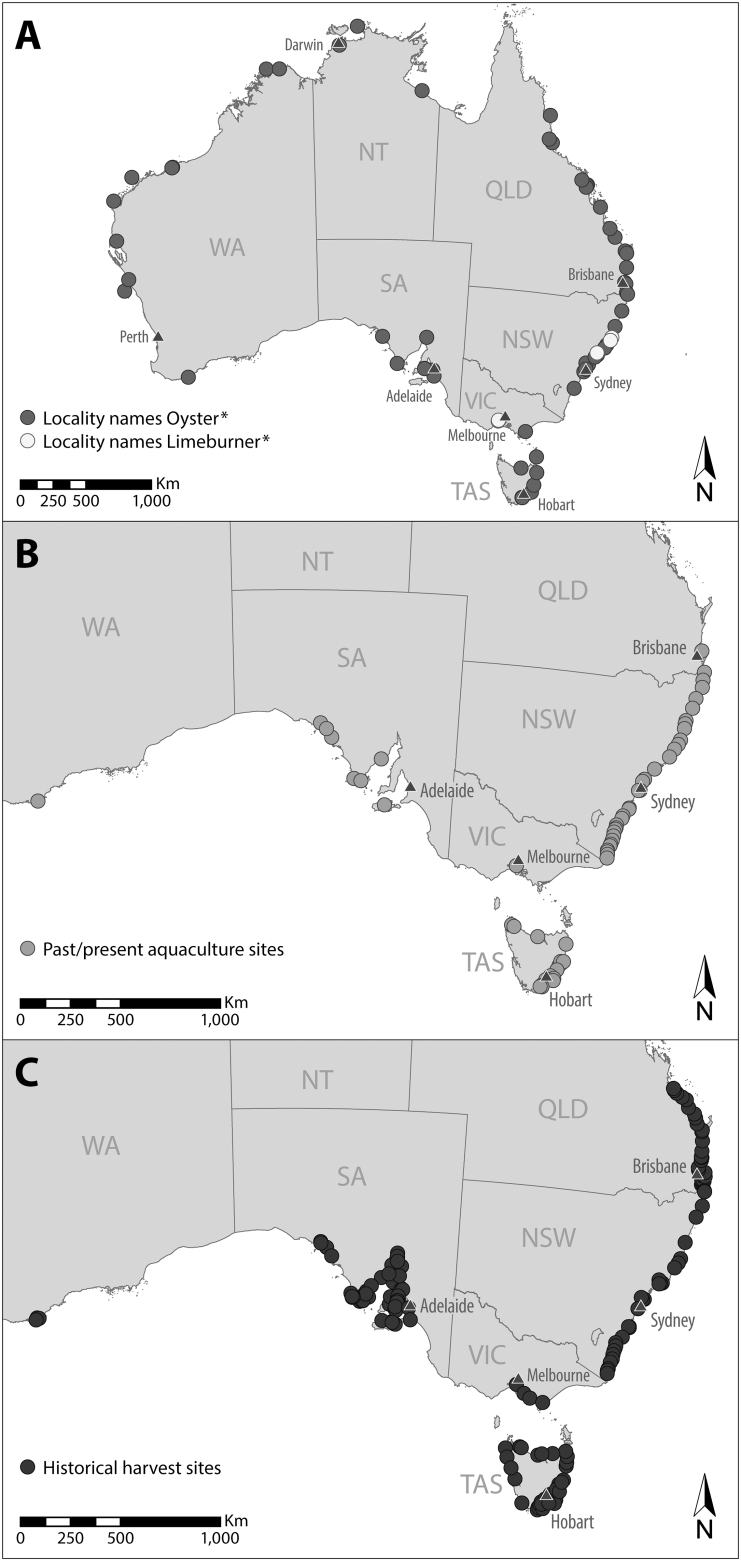
(A) Locality names across Australia containing the term Oyster or Limeburner (B) past and present shellfish aquaculture sites and (C) historical locations of shellfish fisheries.

We identified 59 locations named oyster, five named limeburner and no localities named mussel with location names distributed across Australia (Fig 2A). Sixty past or current shellfish aquaculture sites were also identified across Australia (Fig 2B). For the three commercially harvested species (*O*. *angasi*, *S*. *glomerata* and *M*. *galloprovincialis*) we identified 178 locations where historical fishing once occurred (Fig 2C). Interestingly, many localities with reference to oysters occurred in sparsely populated areas around northern and western Australia in contrast to historical oyster fishing and aquaculture, which occurred predominantly on the more populated eastern and southern coastlines. There were, however, remote areas such as Port Davey in Tasmania and Albany in Western Australia, which had historical oyster fisheries despite their distance from major population centres (e.g. Hobart, Perth) and small population during the 1800s.

### Indigenous use

Shellfish have historically been an important food source for Indigenous Australians [48–51], with shell middens dating back at least 6,000 years [52]. The ecosystem-forming species *O*. *angasi*, *S*. *glomerata*, *S*. *cucullata*, *M*. *galloprovincialis*, and *T*. *hirsuta* were important coastal food sources for Aboriginal communities in pre-European times [53–55] with shells from these species making up large proportions of middens in coastal areas of Australia, including along the Queensland coast and islands [49, 56, 57] in New South Wales [52], Victoria [58], Tasmania [59] and in some parts of South Australia [60]. In southern Western Australia and some areas of South Australia, Indigenous use and consumption of shellfish may have been limited, with patterns of use reflecting consumption of shellfish species that were comparatively easier to forage [60]. In other areas they may have been considered taboo and avoided entirely [60]. Historical accounts from early Europeans further support the reliance of coastal Indigenous Australians on maritime resources [61–65] including farming and trading of *S*. *glomerata* [66, 67].

### Early accounts, colonial use and historical harvest

James Cook provided the first European account of extensive oyster reefs in Botany Bay in 1777 [68]. Several other early explorers also documented extensive reefs in locations such as Port Phillip Bay, Victoria [69] Streaky Bay, South Australia [70] and Oyster Harbour, Western Australia [71]. *O*. *angasi* and *S*. *glomerata* were commercially harvested by dredge and hand methods from first European settlement in Sydney in 1788 [72] and at the same time or shortly after the establishment of Australia’s other major colonies of Hobart (*in 1804*, [73]), Brisbane (*in 1824*, [72]), Melbourne (*in 1835*, [74]) and Adelaide (*in 1836*, [37]). These fisheries expanded to bays and estuaries located farther away from Australia’s first colonies as local resources became depleted and demand increased [7, 37], resulting in a valuable commercial harvest fishery for *O*. *angasi* and *S*. *glomerata*, which spanned much of their distributions.

At its peak the commercial harvest industry provided hundreds of direct jobs in fishing, fish mongering and restaurants (e.g. oyster bars), lime production and boat building [48, 74, 75], whilst also providing a cheap and accessible food source that sustained early Australian coastal and inland colonial expansion for nearly 100 years and coastal Aboriginal communities for thousands of years prior to European colonisation.

### Historical extent and decline

The extent to which all ecosystem-forming species listed in Table 1, with the exception of *C*. *gigas*, M. *galloprovincialis*, *O*. *angasi and S*. *glomerata*, have declined or expanded since European settlement is difficult to quantify, with the prior extent and current ecosystem-forming distribution of these species largely undocumented. We hypothesise the lack of available data on these species are likely a result of a combination of lack of commercial use, their more sporadic ecosystem-forming nature and occurrence in more remote and sparsely populated areas (i.e. northern Australia).

Oyster reefs created by *C*. *gigas* have increased in extent since their introduction for aquaculture from Japan into Tasmania and Western Australia in 1947, and into Victoria in 1953 and South Australia in 1969. The Western Australian populations eventually died out, but *C*. *gigas* successfully established wild populations in all other states. *C*. *gigas* has outcompeted and replaced native intertidal *S*. *glomerata* ecosystems in some NSW locations [76] where it is listed as a Class 2 Noxious Species. Despite being viewed as a threat to native *S*. *glomerata*, wild populations and aquaculture of *C*. *gigas* may have a similar ecosystem value for benthic invertebrate communities as *S*. *glomerata* [77].

Commercial harvest of *M*. *galloprovincialis* does not appear to have been important during colonial times, but appears to have increased with the arrival of eastern European immigrants in the 1950s and 1960s in Victoria [39]. Between 1964 and 2005 over 11,000 tonnes of *M*. *galloprovincialis* were commercially harvested from Port Phillip Bay, Victoria, mostly by dredging. Commercial harvest peaked at around 1000 tonnes per year in the mid-late 1970s and again in the mid-1980s. After 1987, commercial catches were on average less than 1% of peak catches [39] and have since ceased in all locations. Although anecdotal accounts of contemporary *M*. *galloprovincialis* beds exist, no locations with substantial beds are currently known by the relevant authorities in Victoria [P. Hamer, *Pers*. *Comm*.].

**Fig 3:**
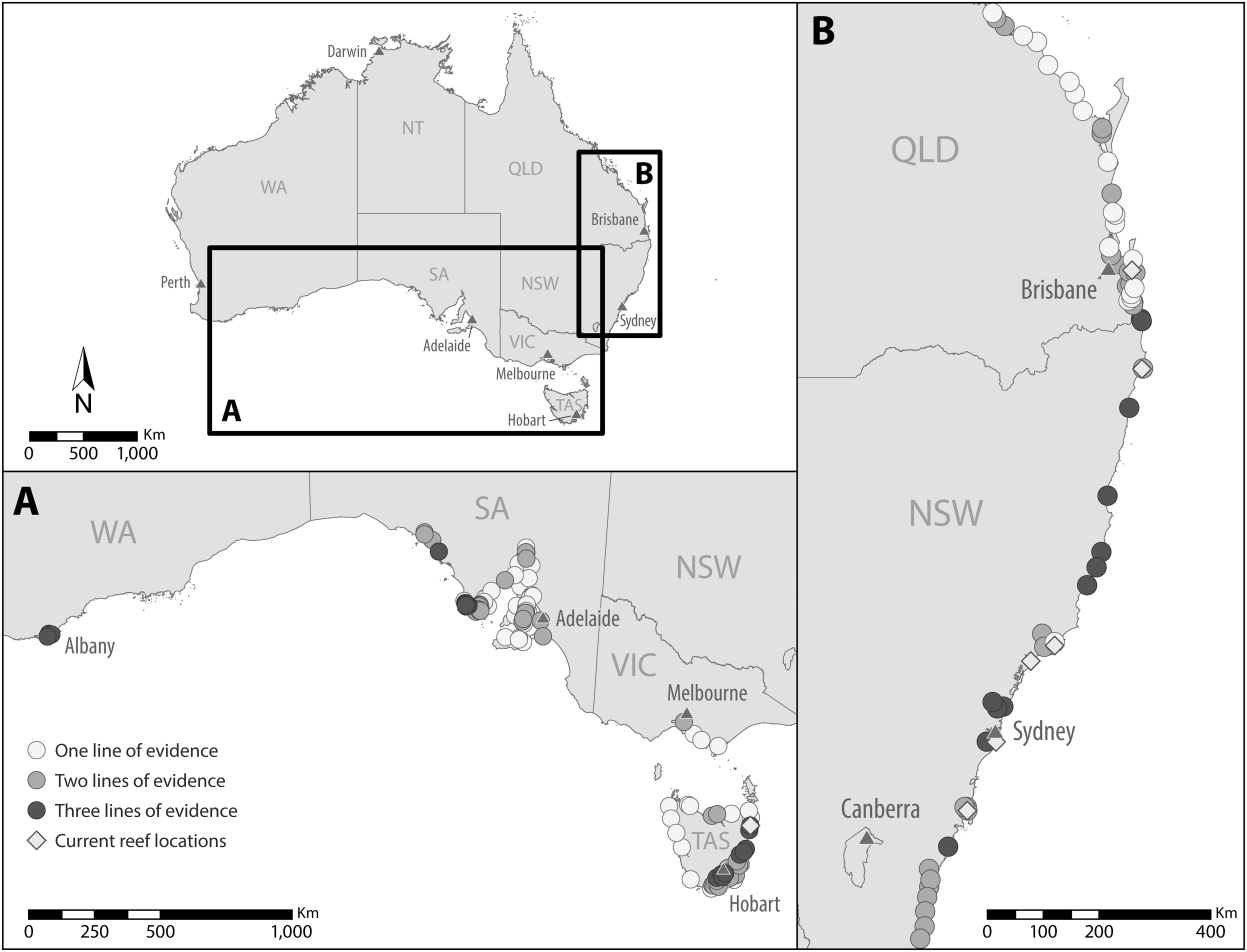
Historical shellfish ecosystem locations deciphered from multiple lines of evidence for: (A) *Ostrea angasi* and (B) *Saccostrea glomerata*. Evidence based on: 1) Historically harvested locations 2) locality names consisting of either ‘Oyster’ or ‘Limeburner’ or 3) current commercial shellfish aquaculture (*S*. *glomerata*, *O*. *angasi* and *C*. *gigas only)*.

Due to the lack of data on population extent for most ecosystem-forming shellfish species, we focus the remainder of this study on the two species for which we could assess historic and current extent, *O*. *angasi* and *S*. *glomerata*. For *O*. *angasi*, 177 locations were identified across its natural distribution, which: 1) were/are commercially harvested for shellfish (118 locations), 2) contained the name ‘Oyster’ or ‘Limeburner’ (33 locations) or 3) were/are being used for shellfish aquaculture (26 locations) (Fig 3A). Eighty-eight (50%) of these locations had two lines of overlapping evidence whilst 20 sites (17%) contained all three lines of evidence. For *S*. *glomerata*, 126 locations were identified across its natural distribution (60 commercially harvested for shellfish, 34 containing the name ‘Oyster’ or ‘Limeburner’ and 35 were/are being used for shellfish aquaculture) (Fig 3B). Fifty-five (42%) of these locations overlapped with two lines of evidence, whilst 13 sites (20%) overlapped with all three lines of evidence. We identified a number of new harvest locations in Western Australia and Tasmania (for *O*. *angasi*) which were previously unreported in the scientific literature, and, collectively, the number of locations in which shellfish ecosystems were likely to have been harvested in commercial quantities (combined for both species) across Australia was likely to have been circa 178 locations.

When viewed collectively, our multiple lines of evidence combined with previous studies indicate that shellfish ecosystems formed by *O*. *angasi* and *S*. *glomerata* were once a common and extensive marine ecosystem in Australia but have since followed a global trend of near total loss [13, 17]. Today, only a single *O*. *angasi* reef system is known to exist that is comparable in size to reef systems historically (commercially) harvested [40], compared to at least 118 previously harvested locations. Out of the 60 historically fished locations identified for *S*. *glomerata*, only six are known to still contain commercially harvestable sized reef systems [42], indicating that less than 1% of *O*. *angasi* reef systems and 8% of *S*. *glomerata* reef systems still remain.

We acknowledge that these estimates do not include reefs systems that may have disappeared from locations which were not fished historically and/or the likelihood that more reef systems are still in existence. Our data are also likely biased towards eastern and southern Australia where the human population is greatest and oyster fishing was most prevalent. Ecosystem-forming species still exist in the majority of locations where historical harvest occurred, and many smaller patches of reefs or beds (i.e. >5 m^2^ but less than 1 ha) of *O*. *angasi* and *S*. *glomerata* are still in existence. Reef and bed patches of unsubstantiated size are also known but not necessarily officially recorded for the other identified species (*authors pers*. *obs*). Knowledge of the existence of current shellfish ecosystem locations are documented largely on an ad hoc basis by estuary managers, shellfish growers, fishers and researchers, hence our approach of using expert elicitation to help substantiate shellfish ecosystem locations where undocumented. We therefore suggest a priority for government agencies and academic institutions should be to identify and classify shellfish ecosystems alongside other ecosystems during assessments to help locate and document existing unknown locations.

Despite considerable amounts of information in the grey literature on the extent of commercial harvest fisheries for ecosystem-forming species, quantitative information on the size and density of shellfish ecosystems was rare and, where available, largely restricted to descriptions of single estuaries covering only a handful of events or at best several years (e.g. 3549 bags (approx. 1.24 M individuals) in South Australia during 1890 [37]; 10 tonnes per week extracted from Western Port (Victoria) during the 1850s [39]; 21,000 sacks (~ 1890 tonnes) in southeast Queensland in 1891 [78] (Table 3). Overall fisheries catch rates were poorly recorded in the early to mid-1800s with most primary sources of data obtained from newspaper articles. This is unlike other countries such as the United States where historical fisheries records are available and have been substantial enough to be used to quantify reef biomass at regional scales [14]. New analyses that can retrospectively provide quantitative estimates of biomass by modelling catch-effort data (e.g. Alleway et al. [79]) may, however, provide future insights into the biomass of historic shellfish ecosystems in Australia.

**Table 3:** Summary of historical wild harvest shellfish fisheries and legislation.

	Tasmania	Queensland (northeast)	Queensland (southeast)	New South Wales	Victoria	South Australia	Western Australia
**Main fishery species**	*Ostrea angasi*	*Saccostrea cucullata*; *Saccostrea glomerata*	*Saccostrea glomerata*	*Saccostrea glomerata*	*Ostrea angasi*; *Mytilus (edulis) galloprovincialis*	*Ostrea angasi*	*Ostrea angasi*
**Evidence of Indigenous use (shell middens, cultural references)**	Yes (*O*. *angasi*; M. *galloprovincialis*) throughout coastal Tasmania	Yes (*S*. *glomerata*; *T*. *hirsute*; *I*. *ephippium*; *S*. *cucullata*; *O*. *angasi*)	Yes (*S*. *glomerata*; *T*. *hirsuta*; Pteriidae spp.)	Yes (*S*. *glomerata*; *O*. *angasi*; *T*. *hirsuta*)	Yes (*O*. *angasi*; *M*. *edulis galloprovincialis*)	Yes (*O*. *angasi*; *M*. *galloprovincialis*)	No, considered taboo
**Number of estuaries/coastal areas with commercial fishery**	16+	11	30 +	21+	4	67	3
**Peak harvest years**	1860–1870	*S*. *cucullata* 1920–1946; *S*. *glomerata* 1870–1920	1860–1910	Up to 1860s	Oysters: 1840–1860, Mussels: 1970–1987	1850 (?)-1900	1850 (?)-1880
**Highest reported number of people employed in single estuary**	Unknown	14	>200 (Moreton Bay)	Unknown	100 (Western Port 1850s)	50	Unknown
**Highest reported number of vessels in single estuary**	17 (double handed boats, Spring Bay)	4 (Mackay 1945)	>127 (Moreton Bay)	64 (Clarence River, 1883)	Oysters: unclear but 100 possibly overall, mussel/scallop fishery in Port Phillip Bay: 80–90 boats 1980s.	25 Cutters Coffin Bay (2 people per boat)	Unknown
**Date of first oyster legislation**	1853 (*Oyster Fisheries Act*, TAS)	1863 (*Oyster Act*, QLD)	1863 (*Oyster Act*, QLD)	1868 (*Oyster Beds Act*, NSW)	1859 (*Oyster Fisheries Act*, VIC)	1853 (*Oyster Beds Act*, SA)	1881 (*Oyster Fisheries Act*, WA)
**Date of first spatial closure**	1853	Unknown	Unknown	Prior to 1864	1859	1873	1881
**Time between first colonial settlement and first oyster legislation**	49 years (Hobart settled in 1804)	30 years (Brisbane settled in 1823)	30 years (Brisbane settled in 1823)	80 years (Sydney settled in 1788)	24 years (Melbourne settled 1835)	17 years (Adelaide settled in 1836)	52 years (Perth settled in 1829)
**Date of first fishery closure**	1908	Never closed	Never closed	1868	1886 Western Port; 1888 Port Albert; 1996 Port Phillip	1895 was recommendation of Inspector to suspend all dredging	Never closed but collapse by 1890
**Highest peak harvest recorded (per year)**	22 million oysters from 5 estuaries	In 1946, 1,500 sacks of oysters (around 135 tonnes) from Rockhampton region	21,000 sacks in 1891 (at 90 kg/sack = 1890 tonnes)	Historical unknown, 1976–77 aquaculture production = 9166 tonnes	Oysters: estimate of 10 tonnes/week in 1850s at Western Port, Mussels: Port Phillip Bay approx. 1000 tonnes in 1975 and in 1986	3549 bags (approx. 1,242,150 individuals) in 1890, believed to be higher prior to these catch statistics	Unknown
**Earliest attempt at restoration**	1885	No attempts known	No attempts known	1883	1860s–1900, many leases granted to attempt cultivation and reseeding, under the *Oyster Act of 1859*	1887	1895
**Number of estuaries/locations with existing shellfish reef(s)**	1	Five known reefs of *I*. *ephippium*. *S*. *cucullata* and *C*. *echinata* still exist in low numbers throughout GBR coastline	Possible remnant *S*. *glomerata* reefs near Dunwich, North Stradbroke Island	4 (varying condition)	Oysters = none, although oysters still present, Mussels = at least Gippsland Lakes, probably some small areas in Port Phillip Bay	0	Unknown

### Causes of decline

Several authors have attributed the primary cause of decline for *O*. *angasi* and *S*. *glomerata* reefs to overexploitation largely through dredge harvesting during the mid to late 19^th^ century [4, 7, 37, 39, 80]. Dredging was analogous to mining, breaking off pieces of reef and removing all size-classes of oysters [50]. Dredging directly removed adult shellfish, and thereby reducing the spawning stock, and also removed, broke up, or buried shell culch material inhibiting spat settlement. Oyster populations were severely reduced in New South Wales estuaries during the 1850s-1870s through a process referred to as skinning, where a schooner would be berthed at an oyster bank at low tide and workers raked together the live oysters to supply lime kilns in Sydney and Newcastle [50]. Reducing the number and extent of oyster reefs or beds within a reef system also likely reduced the resilience of the remaining isolated patches against local impacts such as disease and predation [39]. Increased sediment loads into coastal waters resulting from land use changes could also have contributed to reef burial or covered shell cultch further inhibiting settlement [39]. The removal or burying of hard substrate likely led to complete ecosystem change or phase shift from shellfish ecosystems to soft sediment ecosystems [81] and collapse of the harvest fishery [7, 73].

Invasive animals and new diseases may have also contributed to shellfish ecosystem decline or were at least symptomatic of declining water quality and disturbance. One oyster dredging company undertook a large restocking exercise of *O*. *angasi* (source unknown but possibly New Zealand) in Western Port Bay, Victoria in 1861 [50]. Subsequently, all stocked reefs and surrounding natural reefs died mysteriously in 1862, possibly through the introduction of a new disease (e.g. the parasite *Bonamia* sp.). Ogburn et al. [50] attributed the collapse of subtidal oyster reefs in eastern Australia to the proliferation of spinoid polychaete mud worms in the 1880s, again possibly introduced from New Zealand with live *S*. *glomerata* transport.

Other causes including declining water quality [45, 78] which can precipitate disease [50, 78] are also considered to have contributed to shellfish ecosystem decline or inhibited natural recovery. Many estuaries along the east coast of Australia have been strongly affected by acidification caused by the disturbance of acid-sulphate soils brought about by land use changes and development. Affected water has elevated concentrations of metals and lowered pH, which can lower oyster survival and growth rates [82]. Global patterns of increasing ocean acidification may challenge the recovery of shellfish ecosystems in some locations in the future [83–85].

### Regulation of historical commercial harvest

**Fig 4:**
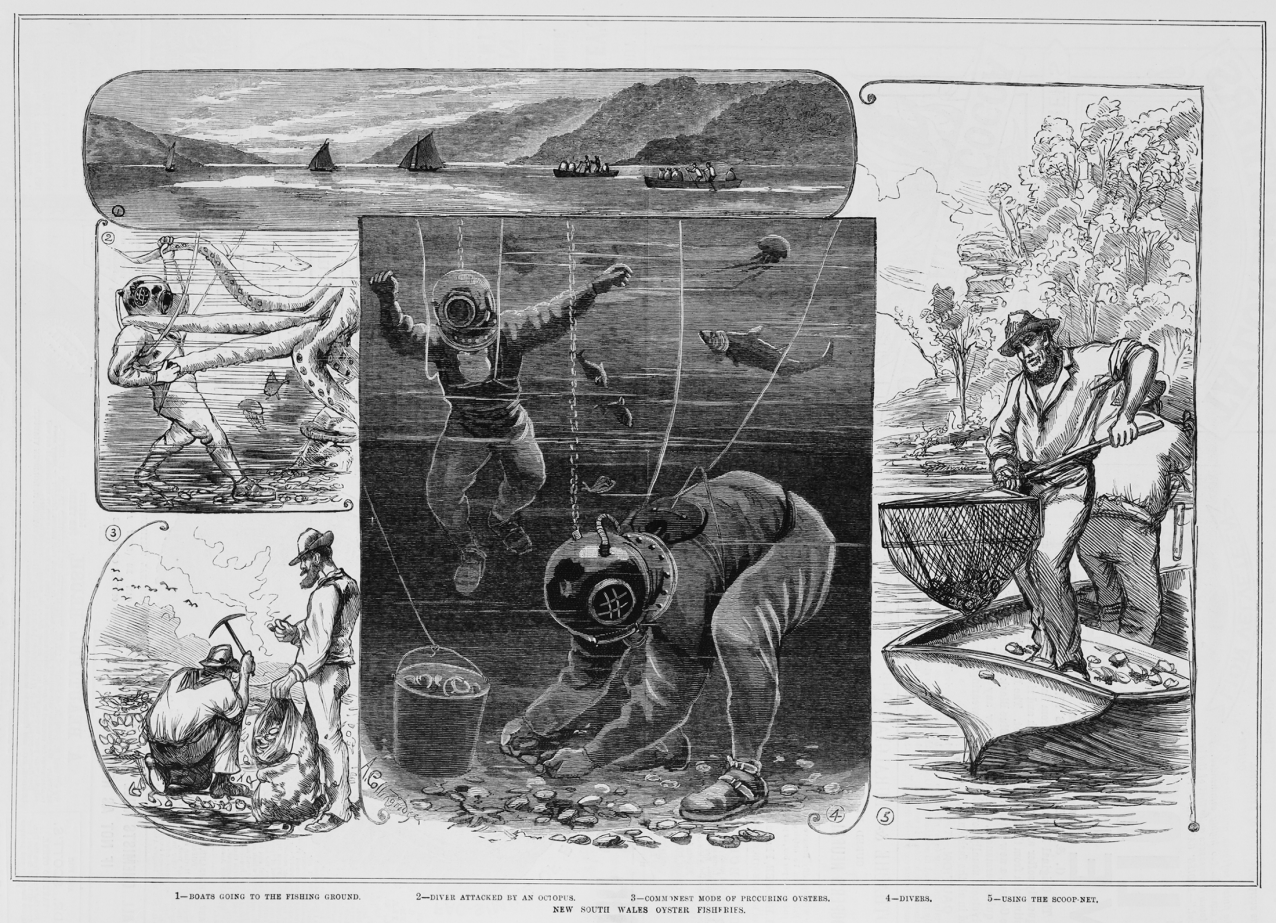
The implementation of oyster harvesting regulation by governments such as the Government of New South Wales in 1868 (Oyster-beds Act) encouraged the establishment of oyster fisheries through propaganda published in popular media. Image 1. Boats going to the fishing ground; Image 2. Diver attacked by an octopus; Image 3. Common mode of procuring oysters; Image 4. *Divers;* Image 5. Using the scope net. Reproduced from [95].

**Fig 5:**
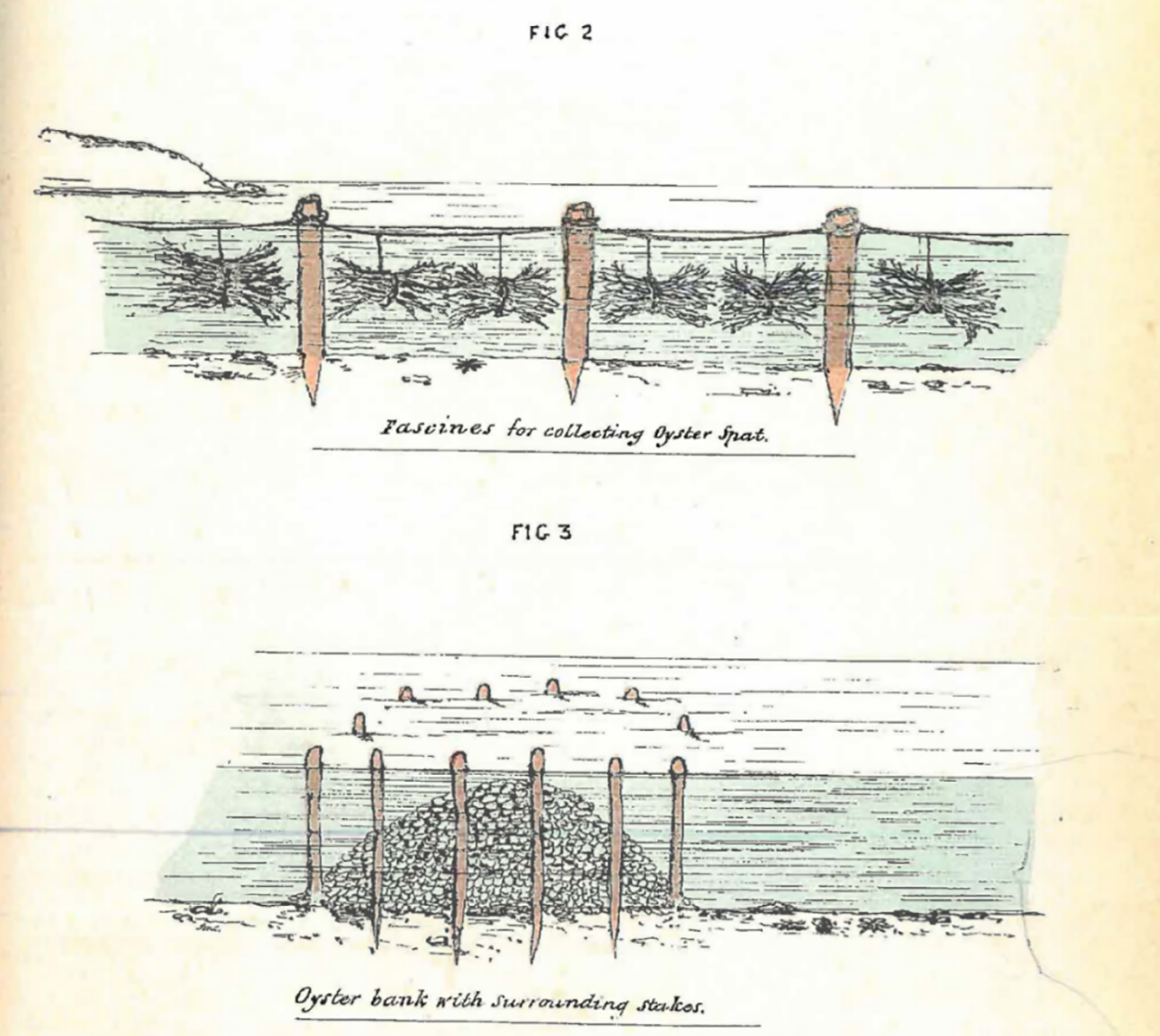
Diagrams created by Saville-Kent (1845–1908) on methods to collect wild oyster spat (Fig 2 in image) and to establish an artificial oyster reef above the seabed (Fig 3 in image). Reproduced from [38].

The commercial harvest of shellfish ecosystems went largely unregulated in each state until concerns about local depletion were raised by fishers and/or fishing regulators which was reported extensively in the media (e.g. The West Australian [86], Brisbane Courier [87], The Queenslander [88], The Mercury [89]). In response to the importance of the oyster fishing industry and to support continued capacity for commercial harvest, state-based regulation of shellfish fisheries occurred from as early as 1853 in South Australia, notably only 17 years since colonial settlement, and in every other Australian state within 80 years of commercial exploitation (Table 3, Fig 4). The oyster industry was the first (of any) fishery to be regulated by legislation in South Australia, Tasmania and Victoria (Table 3). Several reports of historical attempts at replenishing oyster populations in estuaries using primitive aquaculture methods (e.g. adding fresh settlement substrates such as woody debris, (Fig 5) and/or spatial closures have been described for Tasmania [89], South Australia [37], and Western Australia [90]. These efforts were largely through the work of a single individual, English biologist William Saville-Kent (1845–1908), who was appointed as Superintendent and Inspector of Fisheries in Tasmania (1884–1887), Fisheries Consultant for Victoria (1887–1888), Commissioner of Fisheries in Queensland (1889–1892) and Commissioner of Fisheries of Western Australia (1893–1895) [91].

The commercial harvest of wild shellfish ecosystems has since ceased in all Australian states except Tasmania where a small hand harvest industry still exists for *O*. *angasi* [40]. Recreational harvest of oysters and mussels is still permitted in most Australian states with varying daily bag limits (no limit for Queensland or South Australia, 5 litres to 100 individuals across all other states).

### Towards the recovery of Australia’s shellfish ecosystems

The historical exploitation of shellfish ecosystems was an important economic driver for early colonial Australia employing hundreds of people directly in the fishery, shellfish transportation and vessel construction and maintenance. Early fisheries agencies recognised the decline in shellfish harvests and implemented changes in policy and management to reduce overfishing, which included spatial closures, zoning, restoration and, eventually, turning to early forms of aquaculture. In recent times, however, governments and coastal managers have done little to address the protection or restoration of shellfish ecosystems which has, in part, been attributed to the *shifting baseline syndrome* (sensu, Pauly [92]), as a large proportion of the loss occurred in the late 1800s and early 1900s, outside the living memory of most coastal users and managers today [37].

Fortunately, Australia is well equipped to reverse the decline of shellfish ecosystems with a number of existing management and policy tools available to help protect and restore shellfish ecosystems. At the national level mangrove, saltmarsh, seagrass and kelp forest systems are likely to have suffered less loss compared to shellfish ecosystems yet are afforded greater protection under several Commonwealth and State Government conservation legislation (e.g. Commonwealth Government *Environment Protection and Biodiversity Conservation Act* 1999, New South Wales *Threatened Species Conservation Act* 1995 and the Queensland *Fisheries Act* 1994). Considering the extent in decline of *O*. *angasi* and *S*. *glomerata* ecosystems and their current limited distribution and continuing threats to their survival [93] the ecological communities associated with both species would likely qualify for listing under relevant federal and state legislation. Both *O*. *angasi* and *S*. *glomerata* reefs are also likely to qualify for listing under the IUCN Red List of Ecosystems (http://iucnrle.org/).

Australia is a signatory to the Ramsar Convention (which lists shellfish ecosystems as a Ramsar wetland ecosystem type- see Kasoar et al. [38]) and various migratory shorebird international agreements and has one of the largest networks of marine protected areas [94], all of which could be used effectively to encourage the development of local and national recovery plans and protect what’s remaining. However, in order to help enact protection policy and recovery plans, further information on the ecology, function and biodiversity of shellfish ecosystems should be acquired, information that we identified in our literature review as being under-studied.

We therefore recommend the expansion of research into the current distribution, ecology and function of Australia’s shellfish ecosystems in order to inform appropriate levels of conservation, protection and best practice restoration, prioritise resource allocation, as well as strengthen the case for long-term public and private investment in their future management.

### Key actions for Australian coastal marine stakeholders

Knowledge of the loss that has occurred, and its severity, is increasingly motivating restoration works through small to medium-scale on-ground works and research and development trials. Projects are planned in all states (except the Northern Territory) and several have already been implemented with the assistance of private, government and non-government funding. A national network of practitioners, The Shellfish Reef Restoration Network, has been established to help support protection and restoration efforts. Their website (www.shellfishrestoration.org.au) lists many of these restoration activities and provides an online forum for researchers and practitioners to engage and share lessons.

Below we outline three key actions than can help convert knowledge of loss into on-ground restoration and long-term recovery:

1. Raise the profile of shellfish ecosystems by increasing education and communication on their function and value
  - Governments and coastal managers can recognise shellfish ecosystems as a discreet marine ecosystem, which should be included in coastal ecosystem classification, mapping and management processes
  - Educators and community groups can develop communication materials and learning activities such as citizen science, traditional ecological knowledge and oral histories for students, community groups, fishers and Indigenous groups which improve knowledge of shellfish ecosystems
  - Researchers can measure the ecosystem service benefits of Australian shellfish ecosystems to help quantify the benefits of protection and recovery [e.g. 9, 23, 33, 34]
2. Support protection of remaining shellfish ecosystems and eliminate current and future threats by determining eligibility for protection under Commonwealth and State Government threatened ecological community, flora and fauna and fisheries policies and legislation
  - Governments can prioritise and allocate resources for baseline mapping to determine the location, extent and vulnerability of remaining shellfish ecosystems and review the eligibility of shellfish ecosystems under Commonwealth and State Government protection mechanisms
  - Researchers can identify the current extent and biological attributes of remaining shellfish ecosystems to better understand their ecological communities and their vulnerability to threatening processes
  - The community can advocate for greater protection of shellfish ecosystems by recognising their value and supporting government initiatives to protect existing and future shellfish ecosystems
3. Invest in the development of early restoration projects to build momentum, expertise and capacity in Australia’s marine restoration community

Historically-harvested locations could be good candidate sites for restoration efforts, and further research in this area is warranted. However, changes in substrate, water quality and salinity may render historical sites no longer suitable for shellfish ecosystems, and new areas, without historical ecosystems, may now be suitable and could be considered.

- The public and private sector can allocate resources to and partner with shellfish ecosystem restoration projects to facilitate the development of local restoration methods for recovering shellfish ecosystems
- The shellfish aquaculture and commercial fisheries industries can partner with researchers and restoration practitioners to share information and help optimize local restoration methods
- Governments, researchers, communities and the conservation sector can partner with experienced international practitioners and projects to effectively apply best practice shellfish ecosystem restoration methods to Australian projects

## Conclusion

Shellfish ecosystems were once common features in Australia’s estuarine and nearshore marine waters occupying an important ecological position in the marine ecosystem landscape alongside rocky reef, seagrass, mangrove, saltmarsh, coral reef and soft sediment ecosystems. Indigenous Australians and early Europeans benefitted culturally and economically from harvesting and extracting shellfish ecosystems for food and for the production of lime, to the detriment of the shellfish ecosystems and the environmental benefits provided by intact shellfish ecosystems such as fish production, nutrient regulation and coastal protection. Examples from the United States and elsewhere have demonstrated that shellfish ecosystems and their services can be successfully restored. In Australia interest in shellfish ecosystem restoration is increasing with restoration trials starting in most states. As momentum towards shellfish ecosystem restoration continues to grow internationally, Australia could serve as a long-term model for other regions beyond the United States that wish to work towards the future conservation and repair of shellfish ecosystems.

## Supporting information

S1 Table Published research articles and reports on Australian shellfish ecosystem-forming species. See Table 2 for category codes.
(DOCX)

## References

[1] Jones CG, Lawton JH, Shachak M. Organisms as ecosystem engineers. Oikos. 1994; 69(3): 130–47.

[2] Gutiérrez JL, Jones CG, Strayer DL, Iribarne OO. Mollusks as ecosystem engineers: the role of shell production in aquatic habitats. Oikos. 2003;101(1):79–90.

[3] Coen L.D. and Grizzle R., ASMFC, 2007 The Importance of Habitat Created by Shellfish and Shell Beds Along the Atlantic Coast of the U.S., with contributions by Lowery J. and Paynter K.T. Jr., Habitat Management Series #8, 108 pp.

[4] Beck MW B R, Airoldi L, Carranza A, Coen LD, Crawford Defeo CO, Edgar GJ, Hancock B, Kay M, Lenihan H, Luckenbach MW, Toropova CL, Zhang G. Shellfish reefs at risk: a global analysis of problems and solutions. The Nature Conservancy, Arlington VA; 2009.

[5] Todorova V, Micu D, Klisurov L. Unique oyster reefs discovered in the Bulgarian Black Sea. Biologie Hydrobiologie. 2009;62: 871–874.

[6] MacKenzie Jr CL, Burrell Jr VG, Rosenfield A, Hobart WL. The history, present condition, and future of the molluscan fisheries of North and Central America and Europe: Volume 2, Pacific Coast and supplemental topics. US Department of Commerce, NOAA Technical Report 192; 1997.

[7] Kirby MX. Fishing down the coast: historical expansion and collapse of oyster fisheries along continental margins. Proceedings of the National Academy of Sciences of the United States of America. 2004;101(35):13096–9. doi: 10.1073/pnas.0405150101 15326294 10.1073/pnas.0405150101 PMC516522

[8] McLeod IM, Parsons DM, Morrison MA, Le Port A, Taylor RB. Factors affecting the recovery of soft-sediment mussel reefs in the Firth of Thames, New Zealand. Marine and Freshwater Research. 2012;63(1):78–83.

[9] Coen LD, Brumbaugh RD, Bushek D, Grizzle R, Luckenback MW, Posey MH, Powers SP, Tolley SG. Ecosystem services related to oyster restoration. Marine Ecology Progress Series. 2007;341:303–307.

[10] Tolley SG, Volety AK. The role of oysters in habitat use of oyster reefs by resident fishes and decapod crustaceans. Journal of Shellfish Research. 2005;24(4):1007–12.

[11] Dame RF, Zingmark RG, Haskin E. Oyster reefs as processors of estuarine materials. Journal of Experimental Marine Biology and Ecology. 1984;83(3):239–47.

[12] Coen LD, Luckenbach MW. Developing success criteria and goals for evaluating oyster reef restoration: Ecological function or resource exploitation? Ecological engineering. 2000;15(3–4):323–343.

[13] Zu Ermgassen PS, Spalding MD, Blake B, Coen LD, Dumbauld B, Geiger S, et al Historical ecology with real numbers: past and present extent and biomass of an imperilled estuarine habitat. Proceedings of the Royal Society of London B: Biological Sciences. 2012;279(1742):3393–400. 10.1098/rspb.2012.0313 PMC3396889 22696522

[14] Zu Ermgassen PS, Grabowski JH, Gair JR. Quantifying fish and mobile invertebrate production from a threatened nursery habitat. Journal of Applied Ecology. 2015;54:596–606.

[15] Meyer DL, Townsend EC, Thayer GW. Stabilization and erosion control value of oyster cultch for intertidal marsh. Restoration Ecology. 1997;5(1):93–9.

[16] Arkema KK, Guannel G, Verutes G, Wood SA, Guerry A, Ruckelshaus M, et al Coastal habitats shield people and property from sea-level rise and storms. Nature Climate Change. 2013;3:913–918.

[17] Beck MW, Brumbaugh RD, Airoldi L, Carranza A, Coen LD, Crawford C, et al Oyster reefs at risk and recommendations for conservation, restoration, and management. Bioscience. 2011;61(2):107–16.

[18] Waycott M, Duarte CM, Carruthers TJ, Orth RJ, Dennison WC, Olyarnik S, et al Accelerating loss of seagrasses across the globe threatens coastal ecosystems. Proceedings of the National Academy of Sciences. 2009;106(30):12377–81. 10.1073/pnas.0905620106 PMC2707273 19587236

[19] Steneck RS, Graham MH, Bourque BJ, Corbett D, Erlandson JM, Estes JA, et al Kelp forest ecosystems: biodiversity, stability, resilience and future. Environmental conservation. 2002;29(04):436–59.

[20] Connell S, Russell B, Turner D, Shepherd A, Kildea T, Miller D, et al Recovering a lost baseline: missing kelp forests from a metropolitan coast. 2008.

[21] Rogers K, Boon PI, Branigan S, Duke NC, Field CD, Fitzsimons JA, et al The state of legislation and policy protecting Australia’s mangrove and salt marsh and their ecosystem services. Marine Policy. 2016;72:139–55.

[22] Polidoro BA, Carpenter KE, Collins L, Duke NC, Ellison AM, Ellison JC, et al The loss of species: mangrove extinction risk and geographic areas of global concern. PLOS ONE. 2010;5(4):e10095 doi: 10.1371/journal.pone.0010095 20386710 10.1371/journal.pone.0010095 PMC2851656

[23] Zu Ermgassen PM, Hancock B, DeAngelis B, Greene J, Schuster E, Spalding M, et al Setting objectives for oyster habitat restoration using ecosystem services: A manager’s guide. The Nature Conservancy, Arlington VA;2016.

[24] Spalding M, McIvor A, Tonneijck F. Mangroves for coastal defence. Guidelines for coastal managers and policy makers. Wetlands International and The Nature Conservancy; 2015.

[25] Cochard R, Ranamukhaarachchi SL, Shivakoti GP, Shipin OV, Edwards PJ, Seeland KT. The 2004 tsunami in Aceh and Southern Thailand: a review on coastal ecosystems, wave hazards and vulnerability. Perspectives in Plant Ecology, Evolution and Systematics. 2008;10(1):3–40.

[26] Anastas PT, Zimmerman JB. Peer reviewed: design through the 12 principles of green engineering. Environmental science & technology. 2003;37(5):94–101. 10.1021/es032373g 12666905

[27] Crooks S, Herr D, Tamelander J, Laffoley D, Vandever J. Mitigating climate change through restoration and management of coastal wetlands and near-shore marine ecosystems: challenges and opportunities. Environment Department Paper 121, World Bank, Washington DC; 2011.

[28] Aronson J, Alexander S. Ecosystem restoration is now a global priority: time to roll up our sleeves. Restoration Ecology. 2013;21(3):293–6.

[29] Possingham HP, Bode M, Klein CJ. Optimal conservation outcomes require both restoration and protection. Plos Biol. 2015;13(1):e1002052 doi: 10.1371/journal.pbio.1002052 25625277 10.1371/journal.pbio.1002052 PMC4308106

[30] La Peyre M, Furlong J, Brown LA, Piazza BP, Brown K. Oyster reef restoration in the northern Gulf of Mexico: Extent, methods and outcomes. Ocean & Coastal Management. 2014;89:20–8.

[31] Schrack E, Beck M, Brumbaugh R, Crisley K, Hancock B. Restoration works: Highlights from a decade of partnership between The Nature Conservancy and the National Oceanic and Atmospheric Administration’s Restoration Center. The Nature Conservancy, Arlington, VA, USA; 2012.

[32] Kennedy VS, Breitburg DL, Christman MC, Luckenback MW, Paynter K, Kramer K, et al Lessons learned from efforts to restore oyster populations in Maryland and Virginia, 1990 to 2007. Journal of Shellfish Research. 2011;30(3):719–731.

[33] Grabowski JH, Brumbaugh RD, Conrad RF, Keeler AG, Opaluch JJ, Peterson CH, et al Economic valuation of ecosystem services provided by oyster reefs. BioScience. 2012;62(10):900–9.

[34] Edwards P, Sutton-Grier A, Coyle G. Investing in nature: restoring coastal habitat blue infrastructure and green job creation. Marine Policy. 2013;38:65–71.

[35] Gillies CL, Creighton C, McLeod IM. Shellfish reef habitats: a synopsis to underpin the repair and conservation of Australia’s environmentally, socially and economically important bays and estuaries. Report to the National Environmental Science Programme, Marine Biodiversity Hub. Centre for Tropical Water and Aquatic Ecosystem Research (TropWATER) Publication, James Cook University, Townsville; 2015.

[36] Richards RG, Chaloupka M. Using a weight-structured oyster population dynamic model to explore top-down control of coastal water quality in a subtropical embayment. ICES Journal of Marine Science. 2015;72(2) 403–413.

[37] Alleway HK, Connell SD. Loss of an ecological baseline through the eradication of oyster reefs from coastal ecosystems and human memory. Conservation Biology. 2015;29(3):795–804. doi: 10.1111/cobi.12452 25588455 10.1111/cobi.12452

[38] Kasoar T, Zu Ermgassen PS, Carranza A, Hancock B, Spalding M. New opportunities for conservation of a threatened biogenic habitat: a worldwide assessment of knowledge on bivalve-reef representation in marine and coastal Ramsar Sites. Marine and Freshwater Research. 2015;66(11):981–8.

[39] Ford JR, Hamer P. The forgotten shellfish reefs of coastal Victoria: documenting the loss of a marine ecosystem over 200 years since European settlement. Proceedings of the Royal Society of Victoria. 2016;128(1):87–105.

[40] Jones H. and Gardner C. (2016) Small bivalve survey, assessment and stock status update: 2016 Ostrea angasi—Georges Bay Venerupis largillierti—Northern Zone, Georges Bay. Institute for Marine and Antarctic Studies, Hobart Australia. http://www.imas.utas.edu.au/__data/assets/pdf_file/0009/898677/2016_bivalve_assessmentAngasi-and-Venerupis_FINAL.pdf.

[41] Paul LJ. A history of the Firth of Thames dredge fishery for mussels: use and abuse of a coastal resource: New Zealand Aquatic Environment and Biodiversity Report No. 94; 2012.

[42] Fu D, Dunn A, Michael KP, Hills J. The development and performance of a length-based stock assessment of Foveaux Strait oysters (*Ostrea chilensis*, OYU 5) in southern New Zealand, and application to management. Fisheries Research. 2016; 183:506–517.

[43] Sumner C. Oysters and Tasmania. Tasmanian fisheries research. 1972;6(2):1–18.

[44] Nell JA. The history of oyster farming in Australia. Marine Fisheries Review. 2001;63(3):14–25.

[45] Saville-Kent W. Oysters and oyster-culture in Australasia. Australasian Association for Advancement of Science. 1891;3:550–73.

[46] Gillanders BM, Doubleday Z, P Cassey, S Clarke, SD Connell, M Deveney, S Dittmann, et al. Young Spencer Gulf ecosystem & development initiative. Report on scenario development, stakeholder workshops, existing knowledge & information gaps. Report for Spencer Gulf ecosystem and development initiative. The University of Adelaide, Adelaide; 2013.

[47] Butler A, Vicente N, De Gaulejac B. Ecology of the pterioid bivalves *Pinna bicolor* Gmelin and *Pinna nobilis* L. Marine Life. 1993;3(1–2):37–45.

[48] Smith G. The Queensland oyster fishery: An illustrated history: Queensland Department of Primary Industry; 1985.

[49] Ulm S. Coastal themes: an archaeology of the Southern Curtis Coast, Queensland: ANU E Press; 2006.

[50] Ogburn DM, White I, Mcphee DP. The disappearance of oyster reefs from eastern Australian estuaries-impact of colonial settlement or mudworm invasion? Coastal Management. 2007;35(2–3):271–87.

[51] Ross A. Members of the Quandamooka Land Council 1996 Aboriginal approaches to cultural heritage management: A Quandamooka case study. Australian Archaeology ‘95: Proceedings of the 1995 Australian Archaeology Association Annual Conference; 1995.

[52] Stockton ED. Middens of the Central Coast, New South Wales. Australian Archaeology. 1977;(7):20–31.

[53] Meehan B. Shell bed to shell midden: Australian Institute of Aboriginal Studies; Atlantic Highlands, N.J, distributed by Humanities Press; 1982.

[54] Creighton C. Keppel Islands environmental survey: a baseline for archaeological reconstructions and resource management: Archaeology Branch, Department of Community Services; 1984.

[55] Rowland M. The Whitsunday Islands: initial historical and archaeological observations and implications for future work. Queensland Archaeological Research. 1986;3:72–87.

[56] Ulm S. The Seven Mile Creek Mound: new evidence for mid-Holocene Aboriginal marine resource exploitation in central Queensland. Proceedings of the Royal Society of Queensland. 2002;110:121.

[57] Ulm S, Vale D. The antiquity of marine fishing in southeast Queensland: new evidence for pre-2000 BP fishing from three sites on the southern Curtis Coast. An Archaeological Life: Papers in Honour of Jay Hall: Aboriginal and Torres Strait Islander Studies Unit, the University of Queensland; 2006.

[58] Godfrey M. Shell midden chronology in southwestern Victoria: reflections of change in prehistoric population and subsistence? Archaeology in Oceania. 1989;24(2):65–9.

[59] Lourandos H. Dispersal of activities: the east Tasmanian Aboriginal sites. Papers and proceedings of the Royal Society of Tasmania. 1968;102(2):40–46.

[60] Luebbers RA. Meals and menus: a study of change in prehistoric coastal settlements in South Australia. PhD. Thesis, Australian National University. 1978. https://openresearch-repository.anu.edu.au/handle/1885/11040.

[61] Campbell JB. Settlement patterns on offshore islands in Northeastern Queensland. Australian Archaeology. 1979;(9):18–32.

[62] Horrigan B, Young S. Commercial Implications of Native Title. Leichhardt, NSW Brisbane Federation Press in association with the Centre for Commercial and Property Law, Queensland University of Technology; 1997.

[63] Hall J. Sitting on the crop of the bay: An historical and archaeological sketch of Aboriginal settlement and subsistence in Moreton Bay, southeast Queensland. In Bower S. Coastal Archaeology in Eastern Australia: Proceedings of the 1980 Valla Conference on Australian Prehistory. Department of Prehistory, Research School of Pacific Studies, ANU. 1982:79–95.

[64] Hall J. Fishing with Dolphins: Affirming a Traditional Aboriginal Fishing Story in Moreton Bay, SE Queensland In Coleman RJ, Covacevich J, Davie P. Focus on Stradbroke. Boolarong Publications, Brisbane; 1984.

[65] Sullivan M. Ninety years later: a re‐survey of shell middens on Wagonga Inlet and Pambula Lake, NSW. Archaeology in Oceania. 1981;16(2):81–6.

[66] Ross A. Aboriginal approaches to cultural heritage management: A Quandamooka case study. In: Ross A, Ulm S, Lilley IA Australian Archaeology ‘95 –Proceedings of the 1995 Australian Archaeological Association Annual Conference. Australian Archaeological Association, The University of Queensland;1996:107–112.

[67] Kerkhove R. Aboriginal trade in fish and seafoods to settlers in nineteenth-century south-east Queensland: a vibrant industry? Queensland Review. 2013;20(02):144–56.

[68] Attenbrow V. Sydney’s Aboriginal past: investigating the archaeological and historical records: UNSW Press; 2010.

[69] Flinders M. Matthew Flinders-Journal on the Investigator, July 1802-June 1803 (Vol. 2) Matthew Flinders Electronic Archive S. 1801;1:25.

[70] Eyre EJ. Journal of Expeditions of Discovery into Central Australia and Overland from Adelaide to King George Sound in the Years 1840–1 Including an Account of the Manners and Customs of the Aborigines and the State of Their Relations with Europeans: T. W. Boone; 1845.

[71] Vancouver G, Vancouver J. A voyage of discovery to the North Pacific Ocean: and round the world; in which the coast of north-west America has been carefully examined and accurately surveyed: Printed for GG and J. Robinson; 1798.

[72] Smith GS. Southern Queensland’s oyster industry. Journal of the Royal Historical Society of Queensland. 1981;11(3):45–58.

[73] Crawford, C. Protection and repair of Australia’s shellfish reefs- Tasmania Report. Report prepared for the National Environmental Science Program, Australia. https://research.jcu.edu.au/tropwater/research-programs/coastal-estuarine-ecology/shellfish-reef-protection-and-repair/reports-publications

[74] Hannan H B B. Western Port Fisherman. Melbourne, Victoria Hannan and Bennet; 2010.

[75] Shefi D. The development of cutters in relation to the South Australian oyster industry: an amalgamation of two parallel developing industries: Flinders University; 2006.

[76] Bishop MJ, Krassoi FR, McPherson RG, Brown KR, Summerhayes SA, Wilkie EM, et al Change in wild-oyster assemblages of Port Stephens, NSW, Australia, since commencement of non-native Pacific oyster (*Crassostrea gigas*) aquaculture. Marine and Freshwater Research. 2010;61(6):714–23

[77] Wilkie EM, Bishop MJ, O’Connor WA. Are native *Saccostrea glomerata* and invasive *Crassostrea gigas* oysters’ habitat equivalents for epibenthic communities in south-eastern Australia?. Journal of Experimental Marine Biology and Ecology. 2012 6 1;420:16–25.

[78] Diggles B. Historical epidemiology indicates water quality decline drives loss of oyster (*Saccostrea glomerata*) reefs in Moreton Bay, Australia. New Zealand Journal of Marine and Freshwater Research. 2013;47(4):561–81.

[79] Alleway H. K., Thurstan R. H., Lauer P. R., & Connell S. D. (2016). Incorporating historical data into aquaculture planning. ICES Journal of Marine Science: Journal du Conseil, 73(5), 1427–1436.

[80] Hamer P, Pearce B, Winstanley R. Towards Reconstruction of the Lost Shellfish Reefs of Port Phillip Bay. Department of Environment and Primary Industries, Melbourne 2013.

[81] Edgar G, Samson C. Catastrophic decline in mollusc diversity in eastern Tasmania and its concurrence with shellfish fisheries. Conservation Biology. 2004;18(6):1579–88.

[82] Dove MC, Sammut J. Impacts of estuarine acidification on survival and growth of Sydney rock oysters *Saccostrea glomerata* (Gould, 1850). Journal of Shellfish Research. 2007; 26:519–527.

[83] Barton A, Waldbusser GG, Feely RA, Weisberg SB, Newton JA, Hales B. Impacts of coastal acidification on the Pacific Northwest shellfish industry and adaptation strategies implemented in response. Oceanography. 2017;28(2):146–159.

[84] Waldbusser GG, Hales B, Langdon CJ, Haley BA, Schrader P, Brunner EL et al Ocean acidification has multiple modes of action on bivalve larvae. PLOS ONE 2015; http://dx.doi.org/10.1371/journal.pone.0128376 10.1371/journal.pone.0128376 PMC4465621 26061095

[85] Watson S-A, Southgate PC, Tyler PA, Peck L. Early larval development of the Sydney rock oyster Saccostrea glomerata under near-future predictions of CO_2_ driven ocean acidification. Journal of Shellfish Research. 2009;28(3):431–437.

[86] Gale. Our Fisheries. The West Australian (Perth, WA: 1879–1954), 1 December, p. 3: 1899. http://trove.nla.gov.au/newspaper/article/3239674?searchTerm=Our%20Fisheries&searchLimits=l-state=Western+Australia|||l-title=30|||l-decade=189

[87] The oyster industry. Ravages of the worm disease. Certain areas seriously affected. Brisbane Courier. 1898. http://trove.nla.gov.au/newspaper/article/3665726?searchTerm=Ravages%20of%20the%20worm%20disease.%20Certain%20areas%20seriously%20affected&searchLimits=l-state=Queensland

[88] Anon. Fisheries, Moreton Bay oysters. Development of the industry, how it is conducted, mammoth cultivation beds. The Queenslander;1906. http://trove.nla.gov.au/newspaper/article/25969298?searchTerm=Development%20of%20the%20industry%2C%20how%20it%20is%20conducted.%20Mammoth%20cultivation%20beds&searchLimits=l-state=Queensland|||l-title=42

[89] Anon. Commissioners of Fisheries. The Mercury, 14 August 1896. http://trove.nla.gov.au/newspaper/article/9379601?searchTerm=oyster%2C%20closed&searchLimits=l-state=Tasmania|||l-decade=189|||l-year=1896

[90] Saville-Kent W. ‘Albany Oysters Mr Saville-Kent’s Report.’. The West Australian, 28 December 1893. http://trove.nla.gov.au/newspaper/article/3056333?searchTerm=Albany%20Oysters%20Mr%20Saville-Kent%27s%20Report&searchLimits=

[91] Harrison AJ. The Fisheries Savant: William Saville-Kent in Victoria, 1887–8. Historical records of Australian science. 1997;11(3):419.

[92] Pauly D. Anecdotes and the shifting baseline syndrome of fisheries. Trends in ecology and evolution. 1995;10(10):430 21237093 10.1016/s0169-5347(00)89171-5

[93] Committee State of the Environment. Australia State of the Environment 2011—in brief. Independent report to the Australian Government Minister for Sustainability, Environment, Water. Population and Communities Canberra: Department of Sustainability, Environment, Water, Population and Communities;2011.

[94] Wescott G. and Fitzsimons J. eds. Big, Bold and Blue: Lessons from Australia’s Marine Protected Areas. CSIRO PUBLISHING; 2016.

[95] The Australasian sketcher with pen and pencil. Published by H. George, 1873–1889, Melbourne. Accessed March 2017 from: Trove (http://trove.nla.gov.au).

