# Supplementary material for: Australian shellfish ecosystems: Past distribution, current status and future direction
Source: PLoS One. 2018 Feb 14;13(2):e0190914. doi: 10.1371/journal.pone.0190914 (PMC5812559; doi:10.1371/journal.pone.0190914)
Supplement: S1 Table — See Table 2 for category codes. (DOCX) [file pone.0190914.s001.docx]

| **Article** | **Category code** |
| --- | --- |
| Aladaileh S, Nair SV, Birch D, Raftos DA. Sydney rock oyster (*Saccostrea glomerata*) hemocytes: morphology and function. Journal of invertebrate pathology. 2007 Sep 30;96(1):48-63. | EH |
| Aladaileh S, Rodney P, Nair SV, Raftos DA. Characterization of phenoloxidase activity in Sydney rock oysters (*Saccostrea glomerata*). Comparative Biochemistry and Physiology Part B: Biochemistry and Molecular Biology. 2007 Dec 31;148(4):470-80. | EH |
| Alleway HK, Connell SD. Loss of an ecological baseline through the eradication of oyster reefs from coastal ecosystems and human memory. Conservation Biology. 2015 Jun 1;29(3):795-804. | ND/HI/MK/PT/NPM |
| Amaral V, Cabral HN, Bishop MJ. Moderate acidification affects growth but not survival of 6-month-old oysters. Aquatic Ecology. 2012 Mar 1;46(1):119-27. | BI/TS |
| Amaral V, Thompson EL, Bishop MJ, Raftos DA. The proteomes of Sydney rock oysters vary spatially according to exposure to acid sulfate runoff. Marine and Freshwater Research. 2012 Apr 24;63(4):361-9. | BI/EH/ND |
| Anderson K, Burnell F, Roiko A, Andrew M, O’Connor W, Elizur A. Development of a method for identifying elevated vitellogenin gene expression in the Sydney Rock Oyster (*Saccostrea glomerata*) as an indicator of endocrine disruption on the Sunshine Coast. Ecological Management & Restoration. 2010 Aug 1;11(2):143-6. | EH |
| Anderson MJ, Connell SD. Predation by fish on intertidal oysters. Marine Ecology Progress Series. 1999 Oct 14;187:203-11. | HV/CO/PT |
| Anderson TJ, Wesche S, Lester RJ. Are outbreaks of *Marteilia sydneyi* in Sydney rock oysters, *Saccostrea commercialis*, triggered by a drop in environmental pH? Marine and Freshwater Research. 1994 Jan 1;45(7):1285-7. | EH |
| Andrew MN, O’Connor WA, Dunstan RH, MacFarlane GR. Exposure to 17α-ethynylestradiol causes dose and temporally dependent changes in intersex, females and vitellogenin production in the Sydney rock oyster. Ecotoxicology. 2010 Nov 1;19(8):1440-51. | NR/EH |
| Andrew-Priestley MN, O’Connor WA, Dunstan RH, Van Zwieten L, Tyler T, Kumar A, MacFarlane GR. Estrogen mediated effects in the Sydney rock oyster, *Saccostrea glomerata*, following field exposures to sewage effluent containing estrogenic compounds and activity. Aquatic toxicology. 2012 Sep 15;120:99-108. | EH |
| Appleyard SA, Ward RD. Genetic diversity and effective population size in mass selection lines of Pacific oyster (*Crassostrea gigas*). Aquaculture. 2006 Apr 28;254(1):148-59. | NR/TS |
| Beer AC, Southgate PC. Spat collection, growth and meat yield of *Pinna bicolor* (Gmelin) in suspended culture in northern Australia. Aquaculture. 2006 Aug 31;258(1):424-9. | NR |
| Babarro JM, Comeau LA. Byssus attachment strength of two mytilids in mono-specific and mixed-species mussel beds. Biofouling. 2014 Sep 14;30(8):975-85. | NE/BD |
| Baghurst BC, Mitchell JG. Sex‐specific growth and condition of the Pacific oyster (*Crassostrea gigas* Thunberg). Aquaculture Research. 2002 Dec 1;33(15):1253-63. | CM/TS |
| Bailey GN. The role of molluscs in coastal economies: the results of midden analysis in Australia. Journal of Archaeological Science. 1975 Mar 31;2(1):45-62. | ND/HI |
| Banks SC, Piggott MP, Raftos DA, Beheregaray LB. Microsatellite markers for the Sydney rock oyster, *Saccostrea glomerata*, a commercially important bivalve in southeastern Australia. Molecular Ecology Notes. 2006 Sep 1;6(3):856-8. | EH/TS |
| Batley GE, Fuhua C, Brockbank CI, Flegg KJ. Accumulation of tributyltin by the Sydney rock oyster, *Saccostrea commercialis*. Marine and Freshwater Research. 1989 Jan 1;40(1):49-54. | EH/ND |
| Bayne BL, Svensson S. Seasonal variability in feeding behaviour, metabolic rates and carbon and nitrogen balances in the Sydney oyster, *Saccostrea glomerata* (Gould). Journal of experimental marine biology and ecology. 2006 May 2;332(1):12-26. | FE/TS |
| Birch GF, Apostolatos C, Taylor SE. A remarkable recovery in the Sydney rock oyster (*Saccostrea glomerata*) population in a highly urbanised estuary (Sydney estuary, Australia). Journal of Coastal Research. 2013 Mar 19;29(5):1009-15. | ND/NPM |
| Birch GF, Apostolatos C. Use of sedimentary metals to predict metal concentrations in black mussel (*Mytilus galloprovincialis*) tissue and risk to human health (Sydney estuary, Australia). Environmental Science and Pollution Research. 2013 Aug 1;20(8):5481-91. | EH/ND |
| Birch GF, Scammell MS, Besley CH. The recovery of oyster (*Saccostrea glomerata*) populations in Sydney estuary (Australia). Environmental Science and Pollution Research. 2014 Jan 1;21(1):766-73. | ND/NPM |
| Bishop MJ, Krassoi FR, McPherson RG, Brown KR, Summerhayes SA, Wilkie EM, O'Connor WA. Change in wild-oyster assemblages of Port Stephens, NSW, Australia, since commencement of non-native Pacific oyster (*Crassostrea gigas*) aquaculture. Marine and Freshwater Research. 2010 Jul 16;61(6):714-23 | HV/ND/IS |
| Black R, Peterson CH. Biological vs. physical explanations for the non-random pattern of host occupation by a macroalga attaching to infaunal bivalve molluscs. Oecologia. 1987 Sep 1;73(2):213-21. | BD |
| Bleaney A, Hickey CW, Stewart M, Scammell M, Senjen R. Preliminary investigations of toxicity in the Georges Bay catchment, Tasmania, Australia. International Journal of Environmental Studies. 2015 Jan 2;72(1):1-23. | EH |
| Borsa P, Daguin C, Bierne N. Genomic reticulation indicates mixed ancestry in Southern‐Hemisphere Mytilus spp. mussels. Biological Journal of the Linnean Society. 2007 Dec 1;92(4):747-54. | EH/ND |
| Bourke P. Goyder and the ancient Aboriginal shell mounds of Virginia. Northern Territory Historical Studies. 2014(25):45. | ND/CC/HI |
| Brake F, Ross T, Holds G, Kiermeier A, McLeod C. A survey of Australian oysters for the presence of human noroviruses. Food microbiology. 2014 Dec 31;44:264-70. | EH/IS |
| Brown MR, Kube PD, O'Connor S, Cunningham M, King H. Application of near-infrared reflectance spectroscopy for the rapid chemical analysis of Sydney rock oyster (*Saccostrea Glomerata*) and Pacific oyster (*Crassostrea gigas*). Journal of Shellfish Research. 2012 Dec;31(4):1051-60. | AH |
| Brown MR. Rapid compositional analysis of oysters using visible-near infrared reflectance spectroscopy. Aquaculture. 2011 Jul 4;317(1):233-9. | AH |
| Buroker NE, Hershberger WK, Chew KK. Population genetics of the family Ostreidae. I. Intraspecific studies of *Crassostrea gigas* and *Saccostrea commercialis*. Marine Biology. 1979 Sep 1;54(2):157-69. | ND/IS |
| Burt JS, Ebell GF. Organic pollutants in mussels and sediments of the coastal waters off Perth, Western Australia. Marine Pollution Bulletin. 1995 Nov 30;30(11):723-32. | EH/ND/NPM |
| Buschbaum C, Dittmann S, Hong JS, Hwang IS, Strasser M, Thiel M, Valdivia N, Yoon SP, Reise K. Mytilid mussels: global habitat engineers in coastal sediments. Helgoland Marine Research. 2008 Nov 1;63(1):47. | HV/BI |
| Butler AJ. Ecology of *Pinna bicolor* Gmelin (Mollusca: Bivalvia) in Gulf St Vincent, South Australia: density, reproductive cycle, recruitment, growth and mortality at three sites. Marine and Freshwater Research. 1987;38(6):743-69. | BD/BI/NR |
| Butler AJ. Effect of patchsize on communities of sessile invertebrates in gulf St Vincent, South Australia. Journal of Experimental Marine Biology and Ecology. 1991 Nov 27;153(2):255-80. | BI |
| Butler AJ. Razor fish and scallops In: Shepherd SA, Bryars S, Kirkegaard I, Harbison P, Jennings JT (Eds) Natural history of Gulf St Vincent. Adelaide, S. A.: Royal Society of South Australia. 2008. 238-247. | BD/BI/ND |
| Butler AJ, Brewster FJ. Size distributions and growth of the fan-shell *Pinna bicolor* Gmelin (Mollusca: Eulamellibranchia) in South Australia. Marine and Freshwater Research. 1979;30(1):25-39. | BD/BI/NR |
| Butler A, Vicente N, De Gaulejac B. Ecology of the pterioid bivalves *Pinna bicolor* Gmelin and *Pinna nobilis* L. Marine Life. 1993;3(1-2):37-45. | BD |
| Butt D, Aladaileh S, O'Connor WA, Raftos DA. Effect of starvation on biological factors related to immunological defence in the Sydney rock oyster (*Saccostrea glomerata*). Aquaculture. 2007 Apr 6;264(1):82-91. | EH |
| Butt D, O'Connor SJ, Kuchel R, O'Connor WA, Raftos DA. Effects of the muscle relaxant, magnesium chloride, on the Sydney rock oyster (*Saccostrea glomerata*). Aquaculture. 2008 Mar 31;275(1):342-6. | NR/AH |
| Chapman MG, People J, Blockley D. Intertidal assemblages associated with natural corallina turf and invasive mussel beds. Biodiversity & Conservation. 2005 Jun 1;14(7):1761-76. | HV/BI/ND |
| Cheshuk BW, Purser GJ, Quintana R. Integrated open-water mussel (*Mytilus planulatus*) and Atlantic salmon (*Salmo salar*) culture in Tasmania, Australia. Aquaculture. 2003 Mar 27;218(1):357-78. | CM/CO/AH |
| Cheshire AC, Westphalen G. Assessing the status of temperate reefs in Gulf St Vincent IV: Results of the 1999 survey. A Report to the Environment Protection Agency of South Australia. 2000 Jun 30. | BD |
| Coleman N, Mann TF, Mobley M, Hickman N. *Mytilus edulis planulatus*: an “integrator” of cadmium pollution?. Marine Biology. 1986 Jul 1;92(1):1-5. | EH |
| Colgan DJ. Spatial and temporal variation in the genotypic frequencies of the mussel *Brachidontes rostratus*. Heredity. 1981 Jan 1;46(part 2):197-208. | BD |
| Colgan DJ, Ponder WF. Genetic discrimination of morphologically similar, sympatric species of pearl oysters (*Mollusca: Bivalvia: Pinctada*) in eastern Australia. Marine and Freshwater Research. 2002 Jul 19;53(3):697-709. | ND |
| Condie SA, Mansbridge JV, Hart AM, Andrewartha JR. Transport and recruitment of silver-lip pearl oyster larvae on Australia's North West Shelf. Journal of Shellfish Research. 2006 Apr;25(1):179-85. | ND |
| Crawford C. Environmental management of marine aquaculture in Tasmania, Australia. Aquaculture. 2003 Oct 31;226(1):129-38. | HV/EH/ND |
| Denton GR, Burdon-Jones C. Influence of temperature and salinity on the uptake, distribution and depuration of mercury, cadmium and lead by the black-lip oyster *Saccostrea echinata*. Marine biology. 1981 Nov 1;64(3):317-26. | EH |
| Dégremont L, Garcia C, Allen SK. Genetic improvement for disease resistance in oysters: a review. Journal of invertebrate pathology. 2015 Oct 31;131:226-41. | EH/TS |
| Dias PJ, Fotedar S, Snow M. Characterisation of mussel (Mytilus sp.) populations in Western Australia and evaluation of potential genetic impacts of mussel spat translocation from interstate. Marine and Freshwater Research. 2014 Jun 20;65(6):486-96. | EH/ND |
| Diggles BK. Historical epidemiology indicates water quality decline drives loss of oyster (*Saccostrea glomerata*) reefs in Moreton Bay, Australia. New Zealand Journal of Marine and Freshwater Research. 2013 Dec 1;47(4):561-81. | NR/BD |
| Dinamani P. The morphology of the larval shell of *Saccostrea glomerata* (Gould, 1850) and a comparative study of the larval shell in the genus *Crassostrea Sacco*, 1897 (Ostreidae). Journal of Molluscan Studies. 1976 Apr 1;42(1):95-107. | IS/TS |
| Dix TG. Growth of the native oyster *Ostrea angasi* using raft culture in Tasmania, Australia. Aquaculture. 1980 Feb 29;19(2):109-15. | CM/TS |
| Dove MC, O'Connor WA. Reproductive cycle of Sydney rock oysters, *Saccostrea glomerata* (Gould 1850) selectively bred for faster growth. Aquaculture. 2012 Jan 12;324:218-25. | NR/CM/CO |
| Dove MC, O'Connor WA. Salinity and temperature tolerance of Sydney rock oysters *Saccostrea glomerata* during early ontogeny. Journal of Shellfish Research. 2007 Dec;26(4):939-47. | CM/TS |
| Dove MC, Sammut J, Callinan RB. Identification of Environmental Factors, With Particular Reference to Acid Sulfate Soil Runoff, Causing Production Losses in Sydney Rock Oysters (*Saccostrea glomerata*). Fisheries Research and Development Corporation. 2003; 1996: 285. Available: http://frdc.com.au/research/Final_Reports/1996-285-DLD.pdf | AR/AH/APM |
| Dove MC, Sammut J. Histological and feeding response of Sydney rock oysters, *Saccostrea glomerata*, to acid sulfate soil outflows. Journal of Shellfish Research. 2007 Aug;26(2):509-18. | ND/EH |
| Dutton A, Benkendorff K. Biodiversity Assessment and Monitoring of the Port Stanvac Intertidal Reef. Report to the Adelaide and Mt Lofty Natural Resource Management Board. 2008. Flinders University, Adelaide. | BD |
| Edge KJ, Dafforn KA, Simpson SL, Roach AC, Johnston EL. A biomarker of contaminant exposure is effective in large scale assessment of ten estuaries. Chemosphere. 2014 Apr 30;100:16-26. | EH |
| Edge KJ, Johnston EL, Roach AC, Ringwood AH. Indicators of environmental stress: cellular biomarkers and reproductive responses in the Sydney rock oyster (*Saccostrea glomerata*). Ecotoxicology. 2012 Jul 1;21(5):1415-25. | NR/EH |
| Edmunds M, Hart S, Elias J, Power B. Victorian intertidal reef monitoring program: the reef biota in central Victoria and Port Phillip Bay Marine Sanctuaries. Parks Victoria Technical Series. 2004(11). |  |
| English LJ, Maguire GB, Ward RD. Genetic variation of wild and hatchery populations of the Pacific oyster, *Crassostrea gigas* (Thunberg), in Australia. Aquaculture. 2000 Jul 20;187(3):283-98. | IS/TS |
| English LJ, Nell JA, Maguire GB, Ward RD. Allozyme variation in three generations of selection for whole weight in Sydney rock oysters (*Saccostrea glomerata*). Aquaculture. 2001 Feb 15;193(3):213-25. | TS |
| Fitzpatrick JL, Simmons LW, Evans JP. Complex patterns of multivariate selection on the ejaculate of a broadcast spawning marine invertebrate. Evolution. 2012 Aug 1;66(8):2451-60. | NR/BD |
| Ford JR, Hamer P. The forgotten shellfish reefs of coastal Victoria: documenting the loss of a marine ecosystem over 200 years since European settlement. Proceedings of the Royal Society of Victoria 2016 Aug;128.1: 87-105. | HI |
| Gall ML, Poore AG, Johnston EL. A biomonitor as a measure of an ecologically-significant fraction of metals in an industrialized harbour. Journal of Environmental Monitoring. 2012;14(3):830-8. | HV/EH |
| Gaylard S, Thomas S, Nelson M. An assessment of the current status of bioavailable metal contamination across South Australia using translocated mussels *Mytilus Galloprovincalis*. Transactions of the Royal Society of South Australia. 2011 Jan 1;135(1):39-54. | EH/ND |
| Godwin R, Brown I, Montgomery S, Frusher S, Green T, Ovenden J. Telomere dynamics in the Sydney rock oyster (*Saccostrea glomerata*): an investigation into the effects of age, tissue type, location and time of sampling. Marine biology. 2012 Jan 1;159(1):77-86. | CM/TS |
| Goggin CL. Growth of the hairy mussel, *Trichomya hirsuta* (Lamarck 1819), from eastern Australia. Molluscan Research. 1997 Jan 1;18(1):59-66. | CM/CO/AH/TS/RE |
| Green TJ, Barnes AC. Bacterial diversity of the digestive gland of Sydney rock oysters, *Saccostrea glomerata* infected with the paramyxean parasite, *Marteilia sydneyi*. Journal of applied microbiology. 2010 Aug 1;109(2):613-22. | EH/AR |
| Hand RE, Nell JA, Thompson PA. Studies on triploid oysters in Australia: XIII. Performance of diploid and triploid Sydney rock oyster, *Saccostrea glomerata* (Gould, 1850), progeny from a third generation breeding line. Aquaculture. 2004 Apr 26;233(1):93-107. | TS |
| Hassan MM, Qin JG, Li X. Spermatozeugmata structure and dissociation of the Australian flat oyster *Ostera angasi*: Implications for reproductive strategy. Tissue and Cell. 2016 Jun 30;48(3):152-9. | BD/AH |
| Haynes D, Mosse P, Oswald L. The use of transplanted cultured mussels (*Mytilus edulis*) to monitor pollutants along the Ninety Mile Beach, Victoria, Australia—II. Polychlorinated dibenzo-p-dioxins and dibenzofurans. Marine pollution bulletin. 1995 Dec 31;30(12):834-9. | EH |
| Haynes D, Toohey D. Temporal variation in polychlorinated dibenzo-p-dioxins, dibenzofurans, extractable organohalogens (EOX) and heavy metals in commercially cultured mussels (*Mytilus edulis*) from Port Phillip Bay, Victoria, Australia. Oceanographic Literature Review. 1996;6(43):623. | EH |
| Healy JM, Lester RJ. Sperm ultrastructure in the australian oyster *Saccostrea commercialis* (Iredale & Roughley)(Bivalvia: Ostreoidea). Journal of molluscan studies. 1991 Apr 1;57(2):219-24. | BD |
| Hedge LH, Johnston EL. Colonisation of the non-indigenous Pacific oyster *Crassostrea gigas* determined by predation, size and initial settlement densities. PloS one. 2014 Mar 24;9(3):e90621. | HV/IS |
| Hedge LH, Knott NA, Johnston EL. Dredging related metal bioaccumulation in oysters. Marine Pollution Bulletin. 2009 Jun 30;58(6):832-40. | EH |
| Hedge LH, Leung B, O'Connor WA, Johnston EL. The interacting effects of diversity and propagule pressure on early colonization and population size. Journal of Animal Ecology. 2014 Jan 1;83(1):168-75. | NR/BI |
| Hine PM, Thorne T. A survey of some parasites and diseases of several species of bivalve mollusc in northern Western Australia. Diseases of aquatic organisms. 2000 Feb 24;40(1):67-78. | EH/ND |
| Hine PM, Thorne T. Haplosporidium sp. (*Alveolata: Haplosporidia*) associated with mortalities among rock oysters *Saccostrea cuccullata* in north Western Australia. Diseases of aquatic organisms. 2002 Aug 29;51(2):123-33. | EH/BD |
| Hine PM, Thorne T. Haplosporidium sp. (*Haplosporidia)* in Hatchery-Reared Pearl Oysters, *Pinctada maxima* (Jameson, 1901), in north Western Australia. Journal of Invertebrate Pathology. 1998 Jan 31;71(1):48-52. | BD/AH |
| Hindell JS, Quinn GP. Effects of sewage effluent on the population structure of *Brachidontes rostratus* (Mytilidae) on a temperate intertidal rocky shore. Marine and Freshwater Research. 2000;51(6):543-51. | BD |
| Holliday JE, Allan GL, Frances J, Diver LP. Evaluation of commercially-used collectors for Sydney rock oysters, *Saccostrea commercialis* and Pacific oysters, *Crassostrea gigas*. Aquacultural engineering. 1993 Dec 31;12(2):63-79. | CM/CO/TS |
| Holliday JE, Allan GL, Nell JA. Effects of stocking density on juvenile Sydney rock oysters, *Saccostrea commercialis* (Iredale & Roughley), in cylinders. Aquaculture. 1993 Jan 1;109(1):13-26. | CM/CO |
| Holliday JE, Maguire GB, Nell JA. Optimum stocking density for nursery culture of Sydney rock oysters (*Saccostrea commercialis*). Aquaculture. 1991 Jul 1;96(1):7-16. | CM/AH/TS |
| Honkoop PJ, Bayne BL, Drent J. Flexibility of size of gills and palps in the Sydney rock oyster *Saccostrea glomerata* (Gould, 1850) and the Pacific oyster *Crassostrea gigas* (Thunberg, 1793). Journal of Experimental Marine Biology and Ecology. 2003 Jan 8;282(1):113-33. | BD |
| Honkoop PJ, Bayne BL. Stocking density and growth of the Pacific oyster (*Crassostrea gigas*) and the Sydney rock oyster (*Saccostrea glomerata*) in Port Stephens, Australia. Aquaculture. 2002 Oct 18;213(1):171-86. | CM/CO/TS |
| Honkoop PJ. Physiological costs of reproduction in the Sydney rock oyster *Saccostrea glomerata*. Oecologia. 2003 Apr 1;135(2):176-83. | BD/AH/TS |
| Hook SE, Johnston EL, Nair S, Roach AC, Moncuquet P, Twine NA, Raftos DA. Next generation sequence analysis of the transcriptome of Sydney rock oysters (*Saccostrea glomerata*) exposed to a range of environmental stressors. Marine genomics. 2014 Dec 31;18:109-11. | EH |
| Hurwood DA, Heasman MP, Mather PB. Gene flow, colonisation and demographic history of the flat oyster *Ostrea angasi*. Marine and freshwater research. 2005 Dec 12;56(8):1099-106. | ND |
| In VV, O'Connor W, Dove M, Knibb W. Can genetic diversity be maintained across multiple mass selection lines of Sydney rock oyster, *Saccostrea glomerata* despite loss within each?. Aquaculture. 2016 Mar 1;454:210-6. | CM/AH/TS |
| Jackson AC, Chapman MG, Underwood AJ. Ecological interactions in the provision of habitat by urban development: whelks and engineering by oysters on artificial seawalls. Austral Ecology. 2008 May 1;33(3):307-16. | HV/BI/ND/PT |
| Jackson AC. Biogenic habitat on artificial structures: consequences for an intertidal predator. Marine and Freshwater Research. 2009 Jul 14;60(6):519-28. | HV/BI/ND |
| Jones JB, Creeper J. Diseases of pearl oysters and other molluscs: A Western Australian perspective. Journal of shellfish research. 2006 Apr;25(1):233-8. | EH/AH |
| Jordan MA, Teasdale PR, Dunn RJ, Lee SY. Modelling copper uptake by *Saccostrea glomerata* with diffusive gradients in a thin film measurements. Environmental Chemistry. 2008 Aug 19;5(4):274-80. | EH/AR/APM |
| Kesarcodi-Watson A, Lucas JS, Klumpp DW. Comparative feeding and physiological energetics of diploid and triploid Sydney rock oysters, *Saccostrea commercialis*: I. Effects of oyster size. Aquaculture. 2001 Nov 26;203(1):177-93. | TS |
| Keough MJ. Dynamics of the epifauna of the bivalve *Pinna bicolor*: interactions among recruitment, predation, and competition. Ecology. 1984 Jun 1;65(3):677-88. | BD/BI |
| King MG. Cultivation of the Pacific oyster (*Crassostrea gigas*) in a non-tidal hypersaline pond. Aquaculture. 1977 Jun 30;11(2):123-36. | CM/DE/CO/AH |
| Kirby MX. Fishing down the coast: historical expansion and collapse of oyster fisheries along continental margins. Proceedings of the National Academy of Sciences of the United States of America. 2004 Aug 31;101(35):13096-9. | ND/HI/AR/PT |
| Knuckey RM, Brown MR, Barrett SM, Hallegraeff GM. Isolation of new nanoplanktonic diatom strains and their evaluation as diets for juvenile Pacific oysters (*Crassostrea gigas*). Aquaculture. 2002 Aug 23;211(1):253-74. | CM/FE/AH/TS |
| Kookana RS, Shareef A, Fernandes MB, Hoare S, Gaylard S, Kumar A. Bioconcentration of triclosan and methyl-triclosan in marine mussels (*Mytilus galloprovincialis*) under laboratory conditions and in metropolitan waters of Gulf St Vincent, South Australia. Marine pollution bulletin. 2013 Sep 15;74(1):66-72. | EH/CM/FE/AH |
| Kow F, Yu L, FitzGerald D, Grewal D. Understanding the factors related to the consumers' choices of oysters in Australia: an empirical study. Journal of Foodservice. 2008 Aug 1;19(4):245-53. | MK |
| Krassoi FR, Brown KR, Bishop MJ, Kelaher BP, Summerhayes S. Condition‐specific competition allows coexistence of competitively superior exotic oysters with native oysters. Journal of Animal Ecology. 2008 Jan 1;77(1):5-15. | HV/EH/IS |
| Kwan TN, Bolch CJ. Genetic diversity of culturable Vibrio in an Australian blue mussel *Mytilus galloprovincialis* hatchery. Diseases of aquatic organisms. 2015 Sep;116(1):37-46. | EH |
| Lathlean JA, McWilliam RA, Ayre DJ, Minchinton TE. Biogeographical patterns of rocky shore community structure in south‐east Australia: effects of oceanographic conditions and heat stress. Journal of Biogeography. 2015 Aug 1;42(8):1538-52. | BD |
| Lawley M, Birch D. Exploring Point of Sale Strategies for Improving Seafood Retailing: The Case of the Australian Oyster Industry. Journal of Food Products Marketing. 2016 Jun 9:1-7. | AR/MK |
| Lee JH, Birch GF, Cresswell T, Johansen MP, Adams MS, Simpson SL. Dietary ingestion of fine sediments and microalgae represent the dominant route of exposure and metal accumulation for Sydney rock oyster (*Saccostrea glomerata*): a biokinetic model for zinc. Aquatic Toxicology. 2015 Oct 31;167:46-54. | EH |
| Lee JH, Birch GF, Simpson SL. Metal-contaminated re-suspended sediment particles are a minor metal-uptake route for the Sydney rock oyster (*Saccostrea glomerata*)—A mesocosm study, Sydney Harbour estuary, Australia. Marine pollution bulletin. 2016 Mar 15;104(1):190-7. | EH |
| Lee KM, Krassoi FR, Bishop MJ. Effects of tidal elevation and substrate type on settlement and post settlement mortality of the Sydney rock oyster, *Saccostrea glomerata*, in a mangrove forest and on a rocky shore. Journal of Shellfish Research. 2012 Dec;31(4):1043-50. | HV/BI/ND |
| Leith PB, Haward M. Climate Change Adaptation in the Australian Edible Oyster Industry: an analysis of policy and practice. Adaptation Research Network Marine Biodiversity and Resources 2011 Aug 26. Availble at: http://arnmbr.org/content/images/uploads/OYSTER_REPORT_FINAL_web.pdf | CC/AR/MK/APM |
| Lewis JA, Baran IJ, Carey JM, Fletcher LE. A contaminant in decline: long-term TBT monitoring at a naval base in Western Australia. Australasian Journal of Ecotoxicology. 2010 Jan;16(1):17-34. | EH |
| Lewis SE, Wüst RA, Webster JM, Collins J, Wright SA, Jacobsen G. Rapid relative sea-level fall along north-eastern Australia between 1200 and 800cal. yrBP: An appraisal of the oyster evidence. Marine Geology. 2015 Dec 1;370:20-30. | CC/HI/AR |
| Li Y, Qin JG, Li X, Benkendorff K. Monthly variation of condition index, energy reserves and antibacterial activity in Pacific oysters, *Crassostrea gigas*, in Stansbury (South Australia). Aquaculture. 2009 Jan 7;286(1):64-71. | EH |
| Lincoln-Smith MP, Cooper TF. Combining the use of gradients and reference areas to study bioaccumulation in wild oysters in the Hunter River estuary, New South Wales, Australia. Marine pollution bulletin. 2004 May 31;48(9):873-83. | EH |
| Liu B, Li X. Preliminary studies on cryopreservation of Sydney rock oyster (*Saccostrea glomerata*) larvae. Journal of Shellfish Research. 2008;27(5):1125-8. | CM |
| Liu Y, Kow F, Grewal D, FitzGerald D. Consumer purchase behaviour for oysters: an empirical study in some state capital cities of Australia. International Journal of Consumer Studies. 2006 Jan 1;30(1):85-94. | AR/MK |
| Loose SM, Peschel A, Grebitus C. Quantifying effects of convenience and product packaging on consumer preferences and market share of seafood products: The case of oysters. Food Quality and Preference. 2013 Jun 30;28(2):492-504. | AR/MK |
| Lopez LK, Couture P, Maher WA, Krikowa F, Jolley DF, Davis AR. Response of the hairy mussel *Trichomya hirsuta* to sediment-metal contamination in the presence of a bioturbator. Marine pollution bulletin. 2014 Nov 15;88(1):180-7. | EH/ND |
| Madigan TL, Bott NJ, Torok VA, Percy NJ, Carragher JF, de Barros Lopes MA, Kiermeier A. A microbial spoilage profile of half shell Pacific oysters (*Crassostrea gigas*) and Sydney rock oysters (*Saccostrea glomerata*). Food microbiology. 2014 Apr 30;38:219-27. | NR/ND/AH |
| Maguire GB, Nell JA. History, status and future of oyster culture in Australia. InThe 1rst International Oyster Symposium Proceedings, Tokyo, Japan 2005 Jul (pp. 3-12). | HI/AR |
| Mason CJ, Reid DD, Nell JA. Growth characteristics of Sydney rock oysters *Saccostrea commercialis* in relation to size and temperature. Journal of Experimental Marine Biology and Ecology. 1998 Sep 30;227(2):155-68. | CM/FE/TS |
| McCarthy A, Crawford C, Eriksen R, Ross DJ. Dietary preferences, growth and condition of triploid and diploid Pacific Oysters, Crassostrea gigas (Thunberg), in Little Swanport Estuary, Tasmania, Australia. Aquaculture Research. 2015 Apr 28. doi: 10.1111/are.12776 | FE/TS |
| Melwani AR, Thompson EL, Raftos DA. Differential proteomic response of Sydney rock oysters (*Saccostrea glomerata*) to prolonged environmental stress. Aquatic Toxicology. 2016 Apr 30;173:53-62. | EH/TS |
| Minchinton TE, McKenzie LA. Nutrient enrichment affects recruitment of oysters and barnacles in a mangrove forest. Marine Ecology Progress Series. 2008 Feb 7;354:181-189 | HV/BI/ND |
| Mitchell IM, Crawford CM, Rushton MJ. Flat oyster (*Ostrea angasi*) growth and survival rates at Georges Bay, Tasmania (Australia). Aquaculture. 2000 Dec 5;191(4):309-21. | TS |
| Mitchell IM. In situ biodeposition rates of Pacific oysters (*Crassostrea gigas*) on a marine farm in Southern Tasmania (Australia). Aquaculture. 2006 Jun 30;257(1):194-203. | DE/CO/FE |
| Morton B. Competitive grazers and the predatory whelk *Lepsiella flindersi* (Gastropoda: Muricidae) structure a mussel bed (*Xenostrobus pulex*) on a southwest Australian shore. Journal of molluscan studies. 1999 Nov 1;65(4):435-52. | BD/NR |
| Morton B. Predator—prey Interactions Between *Lepsiella Vinosa* (gastropoda: Muricidae) And *Xenostrobus Inconstans* (bivalvia: Mytilidae) In A Southwest Australian Marsh. Journal of Molluscan Studies. 2004 Aug 1;70(3):237-45. | BD |
| Morton B, Lam K, Slack-Smith S. First report of the European flat oyster *Ostrea edulis*, identified genetically, from Oyster Harbour, Albany, south-western Western Australia. Molluscan Research. 2003 Dec 24;23(3):199-208. | ND |
| Nell JA, Cox E, Smith IR, Maguire GB. Studies on triploid oysters in Australia. I. The farming potential of triploid Sydney rock oysters *Saccostrea commercialis* (Iredale and Roughley). Aquaculture. 1994 Oct 1;126(3):243-55. | TS |
| Nell JA, Maguire GB. Commercialisation of triploid Sydney rock and Pacific oysters. Part 1, Sydney rock oysters. Fisheries Research and Development Corporation. 1998; 1993: 151. Available: http://frdc.com.au/research/Final_Reports/1993-151-DLD.pdf | AR/MK/TS |
| Nell JA, O'Riordan PJ, Ogburn DM. Consumer evaluation of diploid and triploid Pacific oysters subjected to high pressure treatment. Journal of shellfish research. 2006 Dec;25(3):1101-4. | AR/MK/AH/TS |
| Nell JA, Perkins B. Evaluation of the progeny of third‐generation Sydney rock oyster *Saccostrea glomerata* (Gould, 1850) breeding lines for resistance to QX disease *Marteilia sydneyi* and winter mortality *Bonamia roughleyi*. Aquaculture Research. 2006 May 1;37(7):693-700. | CM/AH/TS |
| Nell JA, Smith IR, McPhee CC. The Sydney rock oyster *Saccostrea glomerata* (Gould 1850) breeding program: progress and goals. Aquaculture Research. 2000 Jan 1;31(1):45-9. | CM/CO/AH/TS |
| Nell JA. Farming the Sydney rock oyster (*Saccostrea commercialis*) in Australia. Reviews in Fisheries Science. 1993 Jan 1;1(2):97-120. | CO/AR/MK/PT |
| Nell JA. The history of oyster farming in Australia. Marine Fisheries Review. 2001;63(3):14-25. | HI/BD/AR |
| Newton K, Peters R, Raftos D. Phenoloxidase and QX disease resistance in Sydney rock oysters (*Saccostrea glomerata*). Developmental & Comparative Immunology. 2004 May 17;28(6):565-9. | EH |
| Nguyen D. Assessing genetic diversity in cultured aquatic species: The Sydney Rock Oyster (*Saccostrea glomerata*) stock improvement program as a model. M. Sc. Thesis, Queensland University of Technology. 2009. Available at: http://eprints.qut.edu.au/41460/1/Dien_Nguyen_Thesis.pdf | CM/AR/TS/APM |
| O'Connell LG, James NP. Composition and genesis of temperate, shallow-marine carbonate muds: Spencer Gulf, South Australia. Journal of Sedimentary Research. 2015;85(10):1275-91. | ND |
| O'Connor WA, Dove MC. The changing face of oyster culture in New South Wales, Australia. Journal of Shellfish Research. 2009 Nov 27;28(4):803-11. | AR/MK |
| O’Connor WA, Dove M, Finn B, O’Connor S. Manual for Hatchery Production of Sydney Rock Oysters (*Saccostrea glomerata*). Studies. 2008 Jan;60:196-7. | CM/DE/CO/FE/AR/APM/AH/MK/TS |
| O'Connor WA. Latitudinal variation in reproductive behaviour in the pearl oyster, *Pinctada albina sugillata*. Aquaculture. 2002 Jun 28;209(1):333-45. | HV/NR |
| Ogburn DM, White I, Mcphee DP. The disappearance of oyster reefs from eastern Australian estuaries—impact of colonial settlement or mudworm invasion? Coastal Management. 2007 Mar 16;35(2-3):271-87. | HV/BI/ND/PT/RE |
| Olivier F, Ridd M, Klumpp D. The use of transplanted cultured tropical oysters (*Saccostrea commercialis*) to monitor Cd levels in North Queensland coastal waters (Australia). Marine Pollution Bulletin. 2002 Oct 31;44(10):1051-62. | EH |
| Ompi M. Settlement behaviour and size of mussel larvae from the family Mytilidae (*Brachidontes erosus* (Lamarck, 1819), *Brachidontes rostratus* (Dunker, 1857), *Trichomya hirsutus* (Lamarck, 1819), and *Mytilus galloprovincialis* Lamarck, 1819. Journal of Coastal Development. 2011 May 19;13(3):215-27. | NR |
| O'Sullivan BW. The fertility of the Port Lincoln oyster (*Ostrea angasi* Sowerby) from West Lakes, South Australia. Aquaculture. 1980 Jan 1;19(1):1-1. | CM |
| Parker L, Ross P, Raftos D, Thompson E, O'Connor W. The proteomic response of larvae of the Sydney rock oyster, *Saccostrea glomerata* to elevated p CO2. Australian Zoologist. 2011 Jan 1;35(4):1011-23. | EH/BD |
| Parker LM, O’Connor WA, Raftos DA, Pörtner HO, Ross PM. Persistence of positive carryover effects in the oyster, *Saccostrea glomerata*, following transgenerational exposure to ocean acidification. PloS one. 2015 Jul 6;10(7):e0132276. | TS |
| Parker LM, Ross PM, O’Connor WA. Comparing the effect of elevated pCO_2_ and temperature on the fertilization and early development of two species of oysters. Marine Biology. 2010 Nov 1;157(11):2435-52. | EH/BD |
| Parker LM, Ross PM, O’Connor WA. Populations of the Sydney rock oyster, *Saccostrea glomerata*, vary in response to ocean acidification. Marine Biology. 2011 Mar 1;158(3):689-97. | CM/AH/TS/PT/RE |
| Parker LM, Ross PM, O'Connor WA, Borysko L, Raftos DA, Pörtner HO. Adult exposure influences offspring response to ocean acidification in oysters. Global Change Biology. 2012 Jan 1;18(1):82-92. | EH/AR/TS |
| Parker LM, Ross PM, O'Connor WA. The effect of ocean acidification and temperature on the fertilization and embryonic development of the Sydney rock oyster *Saccostrea glomerata* (Gould 1850). Global Change Biology. 2009 Sep 1;15(9):2123-36. | EH/TS |
| Parry GD, Langdon JS, Huisman JM. Toxic effects of a bloom of the diatom *Rhizosolenia chunii* on shellfish in Port Phillip Bay, Southeastern Australia. Marine Biology. 1989 Jul 1;102(1):25-41. | EH/ND/MK |
| Pass DA, Dybdahl R, Mannion MM. Investigations into the causes of mortality of the pearl oyster, *Pinctada maxima* (Jamson), in western Australia. Aquaculture. 1987 Sep 1;65(2):149-69. | AH |
| Paterson KJ, Schreider MJ, Zimmerman KD. Anthropogenic effects on seston quality and quantity and the growth and survival of Sydney rock oyster (*Saccostrea glomerata*) in two estuaries in NSW, Australia. Aquaculture. 2003 May 1;221(1):407-26. | WF/EH |
| Paul-Pont I, Dhand NK, Whittington RJ. Influence of husbandry practices on OsHV-1 associated mortality of Pacific oysters *Crassostrea gigas.* Aquaculture. 2013 Nov 1;412:202-14. | EH/AH/ND |
| Paul-Pont I, Dhand NK, Whittington RJ. Spatial distribution of mortality in Pacific oysters *Crassostrea gigas*: reflection on mechanisms of OsHV-1 transmission. Diseases of aquatic organisms. 2013 Jul 22;105(2):127-38. | EH/AH/ND |
| Peerzada N, Dickinson C. Heavy metal concentration in oysters from Darwin Harbour. Marine pollution bulletin. 1988 Apr 30;19(4):182-4. | EH |
| Peerzada N, Kozlik E. Seasonal variation of heavy metals in oysters from Darwin Harbor, Northern Territory, Australia. Bulletin of environmental contamination and toxicology. 1992 Jan 1;48(1):31-6. | EH |
| Phillips D. The common mussel *Mytilus edulis* as an indicator of pollution by zinc, cadmium, lead and copper. II. Relationship of metals in the mussel to those discharged by industry. Marine Biology. 1976 Oct 1;38(1):71-80. | EH |
| Phillips DJ. The common mussel *Mytilus edulis* as an indicator of pollution by zinc, cadmium, lead and copper. I. Effects of environmental variables on uptake of metals. Marine Biology. 1976 Oct 1;38(1):59-69. | EH |
| Pierce J, McKay J. On community capitals as we see them through photovoice: Cowell oyster industry in South Australia. Australasian Journal of Environmental Management. 2008 Sep 1;15(3):159-68. | ND/HI/AR/MK |
| Potter MA, Hill BJ. Heat mortality in the Sydney rock oyster, *Saccostrea (Crassostrea) commercialis* and the effectiveness of some control methods. Aquaculture. 1982 Aug 1;29(1-2):101-8. | CC/CM/CO |
| Pufahl PK, James NP. Monospecific Pliocene oyster buildups, Murray Basin, South Australia: brackish water end member of the reef spectrum. Palaeogeography, Palaeoclimatology, Palaeoecology. 2006 Apr 4;233(1):11-33. | ND/HI/CC |
| Richards RG, Chaloupka M. Using a weight-structured oyster population dynamic model to explore top-down control of coastal water quality in a subtropical embayment. ICES Journal of Marine Science: Journal du Conseil. 2015 Jan 1;72(2):403-13. | WF/APM |
| Richardson BJ, Garnham JS, Fabris JG. Trace metal concentrations in mussels (*Mytilus edulis planulatus* L.) transplanted into southern Australian waters. Marine pollution bulletin. 1994 Jun 30;28(6):392-6. | EH |
| Reid RG, Brand DG. Giant kidneys and metal-sequestering nephroliths in the bivalve *Pinna bicolor*, with comparative notes on *Atrina vexillum* (Pinnidae). Journal of experimental marine biology and ecology. 1989 Mar 16;126(2):95-117. | EH |
| Roberts D. A comparative study of *Lasaea australis*, *Vulsella spongiarum*, *Pinna bicolor* and *Donacilla cuneata* (Mollusca; Bivalvia) from Princess Royal Harbour, western Australia. Journal of Molluscan Studies. 1984 Oct 1;50(3):129-36. | NR |
| Rose RA, Baker SB. Larval and spat culture of the Western Australian silver-or goldlip pearl oyster, *Pinctada maxima* Jameson (Mollusca: Pteriidae). Aquaculture. 1994 Sep 15;126(1-2):35-50. | CM/CO/TS |
| Rose RA, Dybdahl RE, Harders S. Reproductive cycle of the Western Australian silverlip pearl oyster, *Pinctada maxima* (Jameson)(Mollusca: Pteriidae). J. Shellfish Res. 1990;9(2):261-72. | NR/BD |
| Roubal FR, Masel J, Lester RJ. Studies on *Marteilia sydneyi*, agent of QX disease in the Sydney rock oyster, *Saccostrea commercialis*, with implications for its life cycle. Marine and Freshwater Research. 1989 Jan 1;40(2):155-67. | HV/EH |
| Rubio A. Winberg P, Kirkendale L, Warner R. Ensuring that the Australian Oyster Industry adapts to a changing climate A natural resource and industry spatial information portal for knowledge action and informed adaptation frameworks. Fisheries Research and Development Corporation. 2013; 2010: 534. Available: http://www.frdc.com.au/research/Final_Reports/2010-534-DLD.pdf | CC/AR/MK/APM |
| Russell S, Sullivan CA, Reichelt-Brushett AJ. Aboriginal consumption of estuarine food resources and potential implications for health through trace metal exposure; a study in gumbaynggirr country, Australia. PloS one. 2015 Jun 22;10(6):e0130689. | ND/HI/AR |
| Sampey A, Fromont J. Patterns in marine community assemblages on continental margins: a faunal and floral synthesis from northern Western Australian atolls. Journal of the Royal Society of Western Australia. 2011 Jun;94:267-84. | BI/EH/PT |
| Schmitz HA, Maher WA, Taylor AM, Krikowa F. Effects of cadmium accumulation from suspended sediments and phytoplankton on the oyster *Saccostrea glomerata*. Aquatic Toxicology. 2015 Mar 31;160:22-30. | EH |
| Schrobback P, Pascoe S, Coglan L. History, status and future of Australia’s native Sydney rock oyster industry. Aquatic Living Resources. 2014 Jul 1;27(3-4):153-65. | HI/AR/MK/APM |
| Schrobback P. Economic analyses of Australia's Sydney rock oyster industry. PhD Thesis, Queensland University of Technology. 2015. Availble at: http://eprints.qut.edu.au/83730/ | HI/AR/MK |
| Shefi D. The development of cutters in relation to the South Australian oyster industry: an amalgamation of two parallel developing industries. M. Sc. Thesis, Flinders University. 2006. Available at: http://www.flinders.edu.au/ehl/fms/archaeology_files/dig_library/theses/DebSheffi2006.pdf | CM/CO/TS |
| Smith IR, Sheridan AK, Nell JA. Evaluation of growing methods for use in a Sydney rock oyster *Saccostrea commercialis* (Iredale and Roughley) selective breeding program. Aquaculture. 1995 Apr 1;131(3):189-95. | TS |
| Smith PT. Risks to human health and estuarine ecology posed by pulling out creosote-treated timber on oyster farms. Aquatic toxicology. 2008 Jan 31;86(2):287-98. | EH/AH/ND |
| Southgate PC, Lee PS. Hatchery rearing of the tropical blacklip oyster *Saccostrea echinata* (Quoy and Gaimard). Aquaculture. 1998 Dec 1;169(3):275-81. | NR/CM |
| Spiers ZB, Gabor M, Fell SA, Carnegie RB, Dove M, Connor WO, Frances J, Go J, Marsh IB, Jenkins C. Longitudinal study of winter mortality disease in Sydney rock oysters *Saccostrea glomerata*. Diseases of aquatic organisms. 2014 Jul 24;110(1-2):151-64. | EH |
| Summerhayes SA, Bishop MJ, Leigh A, Kelaher BP. Effects of oyster death and shell disarticulation on associated communities of epibiota. Journal of Experimental Marine Biology and Ecology. 2009 Oct 31;379(1):60-7. | HV/EH/RE/NPM |
| Summerhayes SA, Kelaher BP, Bishop MJ. Spatial patterns of wild oysters in the Hawkesbury River, NSW, Australia. Journal of Shellfish Research. 2009 Aug;28(3):447-51. | ND/AH/NPM |
| Svane I. An Overview of the Blue Mussel in Southern Australia–A Serial Invader, a Blind Passenger, or Just A Welcome Addition to the Menu? Transactions of the Royal Society of South Australia. 2011 Jan 1;135(2):134-9. | ND/EH |
| Swan AA, Thompson PA, Ward RD. Genotype× environment interactions for weight in Pacific oysters (*Crassostrea gigas*) on five Australian farms. Aquaculture. 2007 May 1;265(1):91-101. | TS |
| Takemura Y, Okutani T. Notes on Animals Attached to the Shells of the Silver-Lip Pearl Oyster, *Pinctada maxima* (Jameson), Collected from the “East” Fishing Ground of the Aratura Sea. 日本水産学会誌. 1955;21(2):92-101. | HV/EH/RE/NPM |
| Talbot V, Chegwidden A. Cadmium and other heavy metal concentrations in selected biota from Cockburn Sound, Western Australia. Marine and Freshwater Research. 1982 Jan 1;33(5):779-88. | EH |
| Talbot V. Mobility and speciation of cadmium and lead in polluted seawater of Port Phillip Bay, Australia: management implications. Journal of coastal research. 1989 Oct 1:755-63. | EH |
| Talbot V. Relationship between lead concentrations in seawater and in the mussel *Mytilus edulis*: a water-quality criterion. Marine Biology. 1987 Dec 1;94(4):557-60. | EH/AR/PT/RE |
| Taylor DA, Nair SV, Thompson EL, Raftos DA. Dose‐dependent effects of metals on gene expression in the Sydney rock oyster, *Saccostrea glomerata*. Environmental toxicology. 2015 Sep 1;30(9):989-98. | EH |
| Taylor DA, Thompson EL, Nair SV, Raftos DA. Differential effects of metal contamination on the transcript expression of immune-and stress-response genes in the Sydney Rock oyster, *Saccostrea glomerata*. Environmental pollution. 2013 Jul 31;178:65-71. | NR/EH |
| Taylor JJ, Southgate PC, Rose RA. Fouling animals and their effect on the growth of silver-lip pearl oysters, Pinctada maxima (Jameson) in suspended culture. Aquaculture. 1997 Jun 15;153(1):31-40. | NR/ND/TS |
| Thompson EL, O'Connor W, Parker L, Ross P, Raftos DA. Differential proteomic responses of selectively bred and wild‐type Sydney rock oyster populations exposed to elevated CO2. Molecular ecology. 2015 Mar 1;24(6):1248-62. | TS |
| Thompson EL, Parker L, Amaral V, Bishop MJ, O'Connor WA, Raftos DA. Wild populations of Sydney rock oysters differ in their proteomic responses to elevated carbon dioxide. Marine and Freshwater Research. 2016 Jan 8. | EH/TS/BD/PT |
| Thompson EL, Taylor DA, Nair SV, Birch G, Coleman R, Raftos DA. Optimal acclimation periods for oysters in laboratory-based experiments. Journal of Molluscan Studies. 2012 Jun 4;78(3):304-307 | CM/CO/AH |
| Thomson JD. Metal concentration changes in growing Pacific oysters, *Crassostrea gigas*, cultivated in Tasmania, Australia. Marine Biology. 1982 Apr 1;67(2):135-42. | HI/CM/CO/FE |
| Thomson JM. The acclimatization and growth of the Pacific oyster (*Gryphaea gigas*) in Australia. Marine and Freshwater Research. 1952;3(1):64-73. | ND/HI/IS/AR |
| Thomson JM. The genera of oysters and the Australian species. Marine and Freshwater Research. 1954;5(1):132-68. | NR/BI/ND/IS |
| Thomson JM. The naturalization of the Pacific oyster in Australia. Marine and Freshwater Research. 1959;10(2):144-9. | ND/HI/IS/AR |
| Tran TK, MacFarlane GR, Kong RY, O’Connor WA, Yu RM. Mechanistic insights into induction of vitellogenin gene expression by estrogens in Sydney rock oysters, *Saccostrea glomerata*. Aquatic Toxicology. 2016 May 31;174:146-58. | BD |
| Tranter DJ. Reproduction in Australian pearl oysters (Lamellibranchia). III. *Pinctada albina* (Lamarck): Breeding season and sexuality. Marine and Freshwater Research. 1958;9(2):191-216. | NR/BD |
| Troup AJ, Cairns SC, Simpson RD. Growth and mortality of sibling triploid and diploid Sydney rock oysters, *Saccostrea glomerata* (Gould), in the Camden Haven River. Aquaculture research. 2005 Aug 1;36(11):1093-103. | ND/TS |
| Tynan S, Dutton A, Eggins S, Opdyke B. Oxygen isotope records of the Australian flat oyster (*Ostrea angasi*) as a potential temperature archive. Marine Geology. 2014 Nov 1;357:195-209. | TS |
| Wallis RL. Some aspects of the thermal tolerance of *Trichomya hirsuta* (Mollusca: Bivalvia) of Eastern Australia. Marine Biology. 1977 Sep 1;43(3):217-23. | EH/RE |
| Wallis RL. Thermal tolerance of *Mytilus edulis* of Eastern Australia. Marine Biology. 1975 Jun 1;30(3):183-91. | EH/RE |
| Ward TJ, Young PC. The depauperation of epifauna on *Pinna bicolor* near a lead smelter, Spencer Gulf, South Australia. Environmental Pollution Series A, Ecological and Biological. 1983 Apr 1;30(4):293-308. | BI |
| Ward S, Quinn GP. Preliminary investigations of the ecology of the intertidal predatory gastropod *Lepsiella vinosa* (Lamarck)(Gastropoda Muricidae). Journal of Molluscan Studies. 1988 Feb;54(1):109-17. | BI |
| Ward RD, English LJ, McGoldrick DJ, Maguire GB, Nell JA, Thompson PA. Genetic improvement of the Pacific oyster *Crassostrea gigas* (Thunberg) in Australia. Aquaculture Research. 2000 Jan 1;31(1):35-44. | IS/TS |
| Ward RD, Thompson PA, Appleyard SA, Swan AA, Kube PD. Sustainable genetic improvement of Pacific oysters in Tasmania and South Australia. Fisheries Research and Development Corporation final report Canberra, Australia. 2005. | CM/DE/CO/FE/AR/APM/AH/MK |
| Wells FE. The marine and estuarine molluscs of the Albany area of Western Australia. Rec, West, Aust. Mus, 1980;8(3):335-357 | NR |
| Westfall KM, Gardner J. Genetic diversity of Southern hemisphere blue mussels (*Bivalvia: Mytilidae*) and the identification of non‐indigenous taxa. Biological Journal of the Linnean Society. 2010 Dec 1;101(4):898-909. | BI/ND/IS/CM/PT/NPM |
| Wilkie EM, Bishop MJ, O'Connor WA, McPherson RG. Status of the Sydney rock oyster in a disease-afflicted estuary: persistence of wild populations despite severe impacts on cultured counterparts. Marine and Freshwater Research. 2013 Apr 8;64(3):267-76. | ND/EH/IS |
| Wilkie EM, Bishop MJ, O'Connor WA. Are native Saccostrea glomerata and invasive Crassostrea gigas oysters' habitat equivalents for epibenthic communities in south-eastern Australia? Journal of Experimental Marine Biology and Ecology. 2012 Jun 1;420:16-25. | HV/ND/IS |
| Wilkie EM, Bishop MJ, O'Connor WA. The density and spatial arrangement of the invasive oyster Crassostrea gigas determines its impact on settlement of native oyster larvae. Ecology and evolution. 2013 Dec 1;3(15):4851-60. | HV/ND/IS/NPM |
| Wilkie EM, Bishop MJ. Differences in shell strength of native and non-native oysters do not extend to size classes that are susceptible to a generalist predator. Marine and Freshwater Research. 2013 Jan 16;63(12):1201-5. | HV/ND/EH/IS |
| Wilson BR, Hodgkin EP. A comparative account of the reproductive cycles of five species of marine mussels (Bivalvia: Mytilidae) in the vicinity of Fremantle, Western Australia. Marine and Freshwater Research. 1967;18(2):175-204. | NR |
| Wisely B, Holliday JE, Bennett B. Experimental deepwater culture of the Sydney Rock Oyster (*Crassostrea commercialis*): V. Commercial raft trials. Aquaculture. 1979 Nov 1;18(3):191-201. | CM/DE/CO |
| Wisely B, Holliday JE, Reid BL. Experimental deepwater culture of the Sydney rock oyster (*Crassostrea commercialis*= *Saccostrea cucullata*): I. Growth of vertical clumps of oysters (‘ren’). Aquaculture. 1979 Feb 1;16(2):127-40. | CM/DE/CO |
| Wooton M, Lye AK. Metal levels in the mussel *Mytilus edulis* collected from estuaries in south-eastern Australia. Marine and Freshwater Research. 1982 Jan 1;33(2):363-7. | EH |
| Wu RS, Shin PK. Transplant experiments on growth and mortality of the fan mussel *Pinna bicolor*. Aquaculture. 1998 Apr 1;163(1):47-62. | NR |
| Yonge CM. Form and habit in species of Malleus (including the" hammer oysters") with comparative observations on *Isognomon isognomon*. Biological Bulletin. 1968 Oct 1;135(2):378-405. | EH/ND/CM/CO/AH |
